# Host DNA depletion methods and genome-centric metagenomics of bovine hindmilk microbiome

**DOI:** 10.1128/msphere.00470-23

**Published:** 2023-12-06

**Authors:** Vinícius da Silva Duarte, Davide Porcellato

**Affiliations:** 1 Faculty of Chemistry, Biotechnology and Food Science, Norwegian University of Life Sciences, Ås, Norway; University of California, Davis, California, USA

**Keywords:** bovine mastitis, host DNA depletion, multiple-displacement amplification, shotgun sequencing, short- and long-read sequencing

## Abstract

Bovine mastitis is a multi-etiological and complex disease, resulting in serious economic consequences for dairy farmers and industry. In recent years, the microbiological evaluation of raw milk has been investigated in-depth using next-generation sequencing approaches such as metataxonomic analysis. Despite this, host DNA is a major concern in the shotgun metagenomic sequencing of microbial communities in milk samples, and it represents a big challenge. In this study, we aimed to evaluate different methods for host DNA depletion and/or microbial DNA enrichment and assess the use of PCR-based whole genome amplification in milk samples with high somatic cell count (SCC) by using short- and long-read sequencing technologies. Our results evidenced that DNA extraction performed differently in terms of host DNA removal, impacting metagenome composition and functional profiles.. Moreover, the ratio of SCC/bacteria ultimately impacts microbial DNA yield, and samples with low SCC (SCC below 100,000 cells/mL) are the most problematic. When milk samples with high SCC (SCC above 200,000 cells/mL) underwent multiple-displacement amplification (MDA), we successfully recovered high-quality metagenome-assembled genomes (MAGs), and long-read sequencing was feasible even for samples with low DNA concentration. By associating MDA and short‐read sequencing, we recovered two times more MAGs than in untreated samples, and an ongoing co‐infection not reported by traditional methods was detected for mastitis pathogen. Overall, this new approach will improve the detection of mastitis-associated microorganisms and make it possible to examine host-microbiome interactions in bovine mastitis.

**IMPORTANCE:**

Next-generation sequencing technologies have been widely used to gain new insights into the diversity of the microbial community of milk samples and dairy products for different purposes such as microbial safety, profiling of starter cultures, and host-microbiome interactions. Milk is a complex food matrix, and additionally, the presence of host nucleic acid sequences is considered a contaminant in untargeted high-throughput sequencing studies. Therefore, genomic‐centric metagenomic studies of milk samples focusing on the health‐disease status in dairy cattle are still scarce, which makes it difficult to evaluate the microbial ecophysiology of bovine hindmilk. This study provides an alternative method for genome-centric metagenome studies applied to hindmilk samples with high somatic cell content, which is indispensable to examining host-microbiome interactions in bovine mastitis.

## INTRODUCTION

Bovine mastitis is a multi-etiological and complex disease of dairy cows with serious economic consequences for dairy farmers and industry. It can lead to reduced milk yield, decreased milk quality, and increased costs associated with treatment and management (1). Mastitis is characterized by inflammation of the mammary parenchyma with consequent physical and chemical changes in the gland tissue and glandular secretions (2). Even though various factors can cause mastitis, most cases are caused by bacteria (3).

Among the main pathogens, microorganisms classified as environmental bacteria such as *Staphylococcus aureus*, *Escherichia coli*, *Streptococcus uberis*, *Streptococcus dysgalactiae*, and *Streptococcus agalactiae* survive in the mammary gland and cause clinical mastitis (CM) and subclinical mastitis, manifesting as an increase in milk somatic cell count (SCC) (4). Although traditionally considered normal skin microbiota, coagulase-negative staphylococci have been implicated in several cases of subclinical mastitis in dairy cattle (5). In this group, *Staphylococcus epidermidis*, *Staphylococcus haemolyticus*, and *Staphylococcus chromogenes* are the most common bacteria associated with mastitis (6).

Traditionally, bovine mastitis has been diagnosed in laboratories using culture-based microbiology methods, but these methods cannot detect low-abundant pathogens or co-infections in subclinical mastitis (7). With the rapid advancement of high-throughput next-generation sequencing (NGS) technology and bioinformatics pipelines, culture-independent techniques have exponentially grown in use over the last few years to overcome some inherent limitations of cultivation methods (8, 9). This has become evident due to the increased number of studies concerning the bovine milk microbiome, especially based on targeted approaches such as metataxonomic analyses (10–13).

Milk is a complex food matrix containing several compounds such as minerals, fat, carbohydrates, and proteins that negatively impact microbial DNA extraction protocols and result in low DNA quantity and integrity. Additionally, bovine somatic cells introduce a substantial amount of undesirable host nucleic acid sequences in untargeted high-throughput sequencing studies. Somatic cells are naturally present in raw milk, and their number per milliliter (SCC) is used as an indicator of udder health and milk quality (14). High SCC in raw milk (> 250,000 cells/mL) is indicative of ongoing infection in the mammary gland and can negatively impact the quality of final dairy products, affect the shelf life and flavor, reduce cheese yield, and increase the risk of foodborne illness (15–17). The presence of somatic cells is especially problematic for milk samples displaying a microbial community with low biomass (18), which makes it challenging to examine host-microbiome interactions in bovine mastitis. Due to this limitation, an amplicon sequencing approach has been used for most milk microbiota studies.

Recently, several commercial kits have become available for depleting the host’s DNA from different niches including nasopharyngeal aspirate, saliva, human milk, and bovine milk (18–21). Regarding milk samples, the scientific literature reports the assessment of protocols considering bulk tanks or mock communities (19, 22). However, these samples commonly have a higher microbial load than those found in hindmilk samples. Bovine hindmilk is the milk cows produce toward the end of a milking session and, therefore, no longer enriched in microorganisms present in the teat canal, which ultimately provides a better picture of the udder microbiome (23).

Traditionally, these evaluations have neglected the use of long-read sequencing technologies, and metagenomic shotgun sequencing is frequently carried out with short reads due to the high-throughput generated and reduced costs (24). Currently, long-read sequencing has numerous potential applications, including improving genome sequence assemblies, resolution of repeat-rich regions, and genome contiguity through hybrid assembly (25). However, long-read sequencing technologies require higher DNA concentrations to provide optimal results.

PCR-based whole genome amplification techniques may be used to overcome the high DNA concentration needed for long-read sequencing. One of these methods involves *in vitro* amplification of DNA templates 10,000-fold via multiple displacement amplification (MDA) with a phi29 DNA polymerase and random primers in an isothermal reaction (30°C for 2–16 h) (26, 27). MDA has been integrated into microbial ecology, especially in single-cell genomics and whole-community genome amplification studies due to its high sensitivity and infrequent misincorporation of bases (26, 28). Moreover, MDA has a relatively low representational bias, especially for amplifications starting at higher than 1 ng of DNA.

The correct identification of pathogens associated with mastitis in milk samples using genome-resolved metagenomics approaches can assist the dairy industry or mastitis laboratories in obtaining information at the strain level to provide a better diagnosis of mastitis etiology and sub-dominant species which are routinely ignored during diagnosis (29, 30). Ultimately, these studies aim to assess and improve animal health as well as ensure the safety and quality of human food.

The goals of this study were (i) to evaluate different methods for host DNA depletion and microbial DNA enrichment from milk, (ii) to evaluate the use of MDA and LongAmp in long-read sequencing, (iii) to evaluate the use of short- and long-read sequencing technologies for libraries prepared from microbial DNA obtained of hindmilk samples with different SCC, and (iv) to assess the use of MDA in metagenomic DNA extracted from hindmilk samples with high somatic cell count.

## RESULTS

### Sample rationale

**Fig 1:**
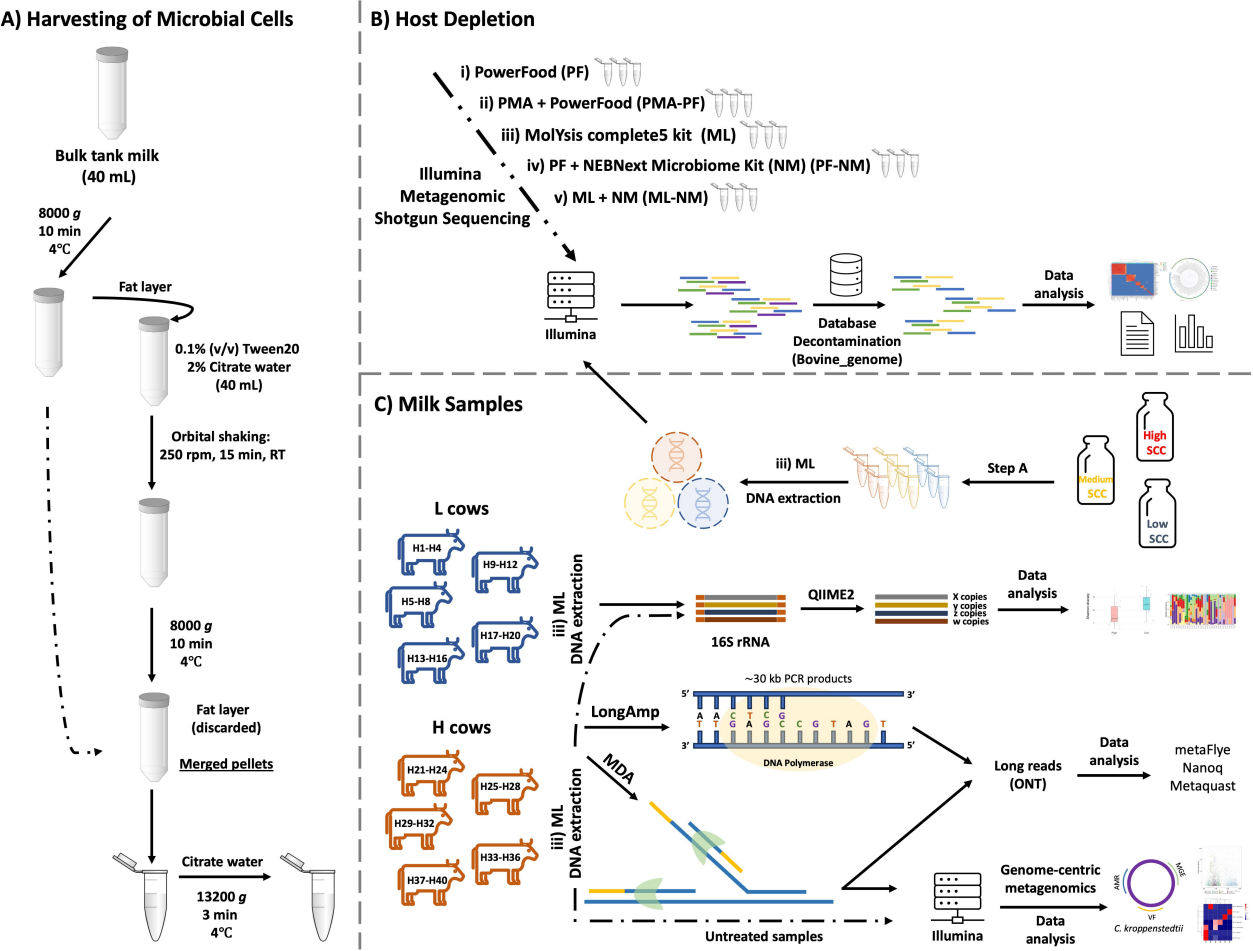
Experimental design. Panels A, B, and C depict the design of each aspect evaluated, with respective numbers of biological and technical replicates. The animals were chosen based on SCC recorded by the automatic milking system (Delaval Online Cell Counter). Five cows with a low SCC (<100,000/mL: quarters H1 to H20) and five cows with a high SCC (>200,000/mL: quarters H22 to H40) were enrolled in this study. Eleven samples (H22, H25, H27, H29, H31, H34, H35, H36, H37, H38, and H39) underwent MDA treatment and were forwarded for metataxonomic analysis. Six out of 11 MDA-treated samples (H22, H25, H29, H31, H34, and H39) were forwarded for shotgun metagenomic sequencing and compared with the corresponding untreated ones.

In this study, we used bulk tank milk (fat: 4.3%; protein: 3.4%; lactose: 4.7%) with a somatic cell count of approximately 125,000 cells/mL and a total bacterial count of 21,000 cells/mL to evaluate the efficiency of bovine DNA removal associated with microbial DNA enrichment by adopting five different protocols consisting of three different commercial kits as described below. The best method was then reevaluated in composite milk from cows with different SCC levels (low, medium, and high). Subsequently, we daily followed up SCC levels of the herd and collected 19 hindmilk samples at the quarter level of five cows with a low SCC (<100,000/mL, quarters H1 to H20) and 20 hindmilk samples at the quarter-level of five dairy cows with high somatic cell count (>200,000/mL, quarters H21 to H40). Microbiological analyses of all quarters revealed an ongoing intramammary infection in 12 out of 39 milk samples. Overall, the major pathogens isolated across the 40 samples and identified by using official mastitis reports (using matrix-assisted laser desorption/ionization-time of flight [MALDI-TOF]) were *Staphylococcus* spp. samples (samples H22, H29, H31, H37, H38, and H39), *Enterococcus* spp. (samples H34, H35, and H36), *Streptococcus* spp. (samples H25 and H27), and *Corynebacterium* spp. (sample H15). According to the individual bacterial count (IBC) obtained from FOSS BactoScan, milk samples collected from infected quarters and with SCC higher than 200,000 cells/mL showed an average bacterial load of 9.2 × 10^5^ cells/mL (coefficient of variation, 5%). Figure 1 depicts the research design and framework adopted in this study.

### Host depletion, microbial enrichment, and DNA extraction

To assess the total DNA concentration, enrichment of microbial reads, and the efficiency of removal of bovine DNA following, we comprehensively evaluated the use of five different methods consisting of two commercial DNA extraction kits and one for enrichment of microbial DNA. The highest DNA yield was observed for the PF (Dneasy PowerFood Microbial Kit) (55.0 ± 5 ng/μL), followed by PF-NM (PF with NEBNext Microbiome DNA Enrichment Kit) (12.4 ± 4.2 ng/μL), ML (MolYsis complete5 Kit) (1.2 ± 0.4 ng/μL), and ML-NM (ML associated with NM) (0.4 ± 0.1 ng/μL). The association PMA-PF yielded a DNA concentration below 0.05 ng/µL. All 15 samples were submitted for library preparation and metagenomic DNA (mgDNA) sequencing; however, due to the low quality of the library produced with the samples extracted with PMA-PF, these samples were not forwarded for shotgun sequencing.

**Fig 2:**
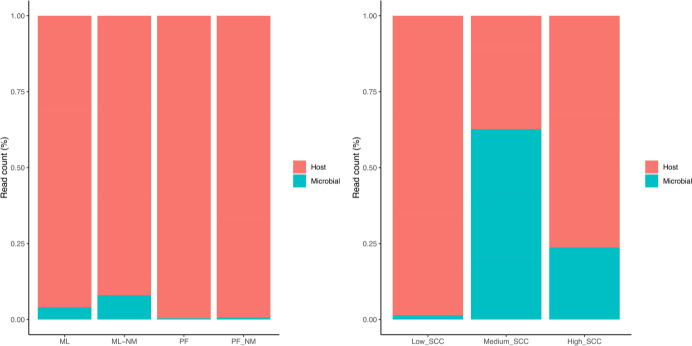
(**A**) Host DNA depletion was evaluated by using four different methods (PF-PMA is not included due to failure during library preparation) applied to bulk tank milk samples. (**B**) Host DNA depletion using ML and applied to milk samples with different levels of somatic cells (high: >200,000 cells/mL; medium: between 100,000 and 200,000 cells/mL; low: <100,000 cells/mL). In total, four different methods were used (*n* = 3/group). ML, *MolYsis* complete5 kit; ML-NM, *MolYsis* complete5 kit performed with NEBNext Microbiome DNA Enrichment kit; PF, DNeasy PowerFood Microbial kit; PF-NM, DNeasy PowerFood Microbial kit performed with NEBNext Microbiome DNA Enrichment kit.

For the first round of sequencing using bulk tank milk, a total of 276.1 million (PF: 20.7 ± 0.6; PF-NM: 27.0 ± 4.5; ML: 21.2 ± 0.9; ML-NM: 23.1 ± 1.8) reads were obtained after trimming and filtering. We adopted bmtagger to perform *in silico* separation of bacterial reads from bovine reads and evaluate the host DNA removal across the five different protocols. Our results indicate that ML-NM efficiently enhanced up to 8.0% of the recovery of microbial reads, whereas ML was around 4.0%. Both methods did not impact the diversity indices. The extraction kits PF-NM and PF yielded only 0.4% and 0.6% of microbial sequences, respectively (Fig. 2A). Based on the similar results found for ML-NM and ML and the higher cost associated with the combination of ML-NM, all subsequent samples were extracted with ML.

We also evaluated the efficiency of removing host DNA from milk samples with low, medium, and high SCC by using ML as an extraction method (n = 3/group). For this analysis, 43.6 million reads were generated (low SCC: 6.8 ± 0.6; medium SCC: 3.6 ± 0.1; high SCC: 3.6 ± 0.7) and evaluated after an initial trimming step and filtering. The best results were achieved when microbial DNA was extracted from samples with medium SCC content (62.7% of the reads; SCC between 100,000 and 200,000 cells/mL), followed by high (23.7% of the reads, SCC higher than 200,000 cells/mL) and low SCC (1.4% of the reads, SCC lower than 10,000 cells/mL), respectively (Fig. 2B).

**Fig 3:**
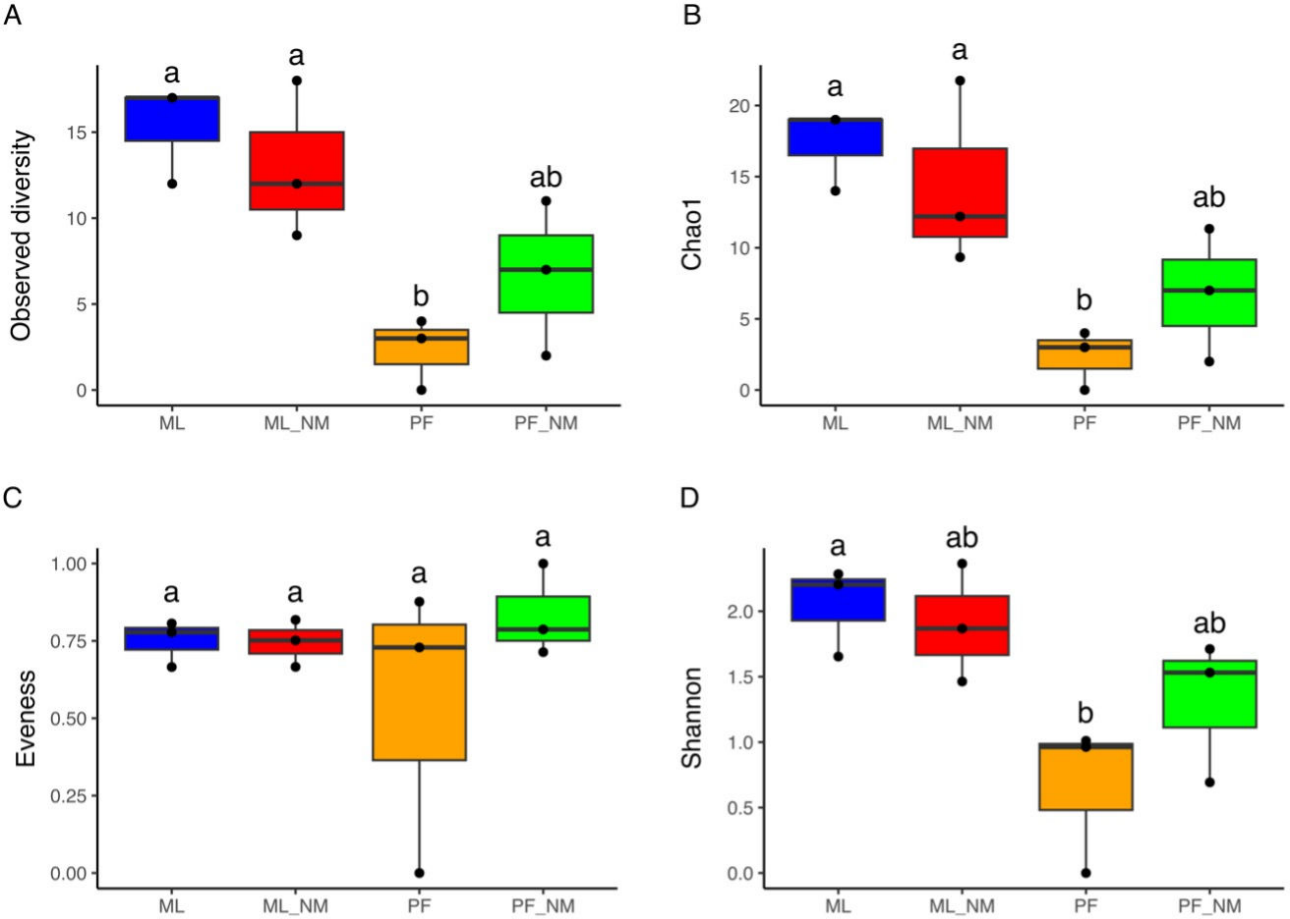
Alpha-diversity indices obtained from different DNA extraction methods after depletion of bovine DNA. Boxes from A to D represent four different alpha-diversity measures. The median value is shown as a line within the box. Whiskers extend to the most extreme value within 1.5 times the interquartile ranges (1.5*IQR). Statistical differences between groups were analyzed by analysis of variance (ANOVA) followed by Tukey’s test (*n* = 3/group). Different letters indicate significant differences between groups. ML, MolYsis complete5 kit; ML-NM, MolYsis complete5 kit performed with NEBNext Microbiome DNA Enrichment kit; PF, DNeasy PowerFood Microbial kit; PF-NM, DNeasy PowerFood Microbial kit performed with NEBNext Microbiome DNA Enrichment kit.

We used MetaPhlAn3 for profiling the composition of the microbial community from metagenomic shotgun sequencing data and for calculating alpha-diversity indices for the different extraction kits. In terms of alpha-diversity indices (Fig. 3), the milk microbial composition of bulk tank milk samples extracted with ML-NM and ML yielded significant differences when compared to PF (*P* < 0.05). The impact of using different kits in the microbial community structure was also investigated by generating matrices of distance based on Bray-Curtis and Jaccard indices. Permutational multivariate analysis of variance using the Adonis function showed that the DNA extraction kit impacted the microbiome composition of the bacterial community (Jaccard: F = 2.9, *P* = 0.005; Bray Curtis: F = 3.7, *P* = 0.004) (Fig. S1). According to pairwise Adonis (Table S1), there was a greater variance between the groups PF vs ML (Jaccard: F.Model = 9.2, R^2^ = 0.7; Bray-Curtis: F.Model = 16.3, R^2^ = 0.8) and PF vs ML_NM (Jaccard: F.Model = 6.6, R^2^ = 0.6; Bray-Curtis = 0.7) than within-group, although not statistically significant (p > 0.05). The lack of significance is attributed to the low sample size used in this study (3 samples/group) and the higher dispersion specially observed for the groups ML-NM and PF-NM.

**Fig 4:**
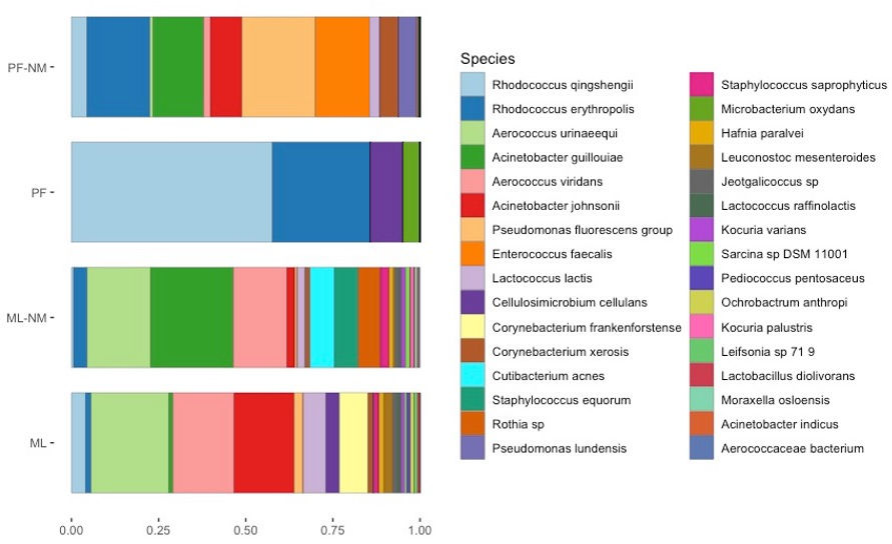
Taxonomical assignment of metagenomics reads with Metaphlan3 after DNA extraction with four different methods (*n* = 3/group). ML, MolYsis complete5 kit; ML-NM, MolYsis complete5 kit performed with NEBNext Microbiome DNA Enrichment kit; PF, DNeasy PowerFood Microbial kit; PF-NM, DNeasy PowerFood Microbial kit performed with NEBNext Microbiome DNA Enrichment kit.

In total, 32 bacterial species were identified across the samples, and the protocols ML-NM and ML were the most efficient in representing the phylotypes commonly present in bulk tank milk (Fig. 4). By analyzing the relative abundance of 10 out of 32 major genera found in bulk milk (e.g., *Staphylococcus, Corynebacterium, Aerococcus, Enterococcus*, and *Pseudomonas*), we observed that there is a significant impact of the different DNA extraction methods in their frequencies, and, overall, ML and ML-NM outperformed the other two methods (Fig. S2). As discussed below, ML employs a chaotropic buffer aiming at the lysis of host cells and the release of eukaryotic DNA, which is subsequently depleted by a DNA-degrading enzyme.

**Fig 5:**
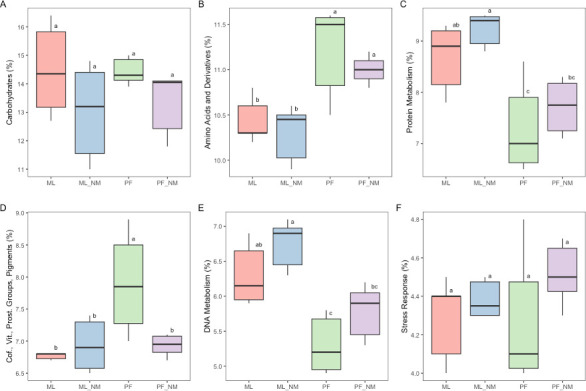
Box plot based on the six most abundant SUPER-FOCUS subsystems (level 1) of the bulk tank milk microbiota after DNA extraction and host depletion methods. The median value is shown as a line within the box. Whiskers extend to the most extreme value within 1.5*IQR. Statistical differences between groups were analyzed by ANOVA followed by Tukey’s test (*n* = 6/group). Different letters indicate significant differences between groups. ML, MolYsis complete5 kit; ML-NM, MolYsis complete5 kit performed with NEBNext Microbiome DNA Enrichment kit; PF, DNeasy PowerFood Microbial kit; PF-NM, DNeasy PowerFood Microbial kit performed with NEBNext Microbiome DNA Enrichment kit.

Lastly, we analyzed the use of different extraction kits and their impact on the microbial community functionality with SUPER-FOCUS. The highest absolute number of reads assigned was obtained from the extraction with the ML-NM kit (6.8 × 10^5^), followed by ML (2.7 × 10^5^), PF-NM (4.6 × 10^4^), and PF (1.8 × 10^4^) (Fig. S3A). Regarding the relative abundance (Fig. S3B), the pairwise comparison revealed that 12 out of 35 subsystems did not differ significantly. However, it is possible to observe that the diverse DNA extraction kits can directly affect the relative abundance of different subsystems and impact housekeeping functions such as amino acids and derivatives, protein metabolism, and DNA metabolism (Fig. 5).

### Long-read metagenomics

#### Oxford Nanopore Technology (ONT) pre-assembly quality assessment

**Fig 6:**
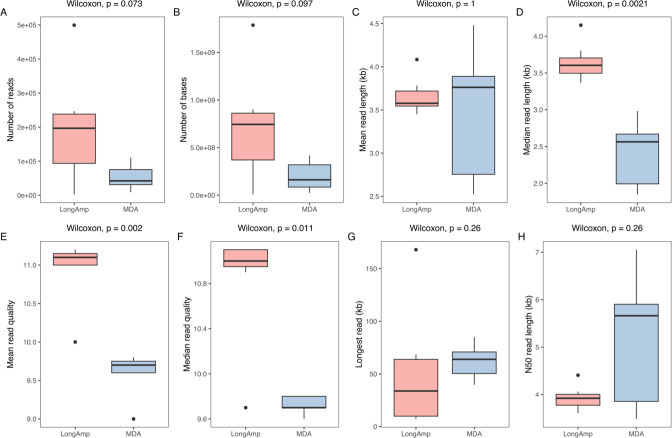
Quality control of long-read sequencing data for milk samples collected from individual quarters with high somatic cell count. The median value is shown as a line within the box. Whiskers extend to the most extreme value within 1.5*IQR. Statistical differences between groups were analyzed with the Wilcoxon test (*n* = 6/group).

We prepared metagenomic DNA with the ML extraction method from six milk samples obtained from quarters where an ongoing intramammary infection (IMI) was recorded. Samples were subjected to PCR and MDA amplification and sequenced with MinION, and the reads were mapped to bovine DNA aiming to remove host sequences from our data set. As depicted in Fig. 6, by adopting the LongAmp protocol, a total of 1.36 × 10^6^ reads were obtained. Mean and median read lengths averaged 3.6 kb, while the longest single read was 168 kb.

Concerning samples amplified with MDA, 3.75 × 10^5^ reads were obtained, with mean and median read lengths of 3.4 and 2.4 kb, respectively. The longest read was 85 kb. Reads that underwent MDA treatment showed a higher N50 than those amplified with LongAmp, although not statistically significant due to a higher coefficient of variation (CV) observed in this group (LongAmp, CV: 6%; MDA, CV: 25%). In terms of read quality, PCR-amplified samples showed a higher and more significant mean/median Q score than those treated with MDA.

#### ONT post-assembly statistics

The taxonomic composition of generated contigs was assessed with the Kraken2 classifier. At the genus level, *Clostridium, Staphylococcus, Enterococcus, Escherichia, Streptococcus, Aerococcus, Pantoea, Corynebacterium, Lactobacillus*, and *Lactiplantibacillus* accounted for the most abundant genera in both groups (Fig. S4). We also noticed an unexpectedly higher relative abundance of sequences assigned to Clostridium in both groups, which might be a misclassification intrinsic to our data set or associated with the classifier of choice for long read. Although differences in the relative abundance of major genera have been observed between the two groups, both groups shared an overall similar bacterial frequency (Fig. S5, *P* > 0.05).

MDA-treated samples showed greater bacterial diversity indices (here evaluated by means of Shannon and evenness, *P* < 0.05) when compared to LongAmp-amplified sequences, but not in terms of observed species and Chao1 (Fig. S6A through D). Microbial functional analysis in MDA- and LongAmp-treated samples was also investigated with SUPER-FOCUS. In total, 33 subsystems were identified and there were no significant differences between both groups (Fig. S7A through AI, *P* > 0.05).

We used MetaQUAST to evaluate the *de novo* assemblies generated with metaFlye on long reads of the six different datasets (H22, H25, H31, H34, H36, and H38) following LongAmp or MDA amplification using ONT reads. As samples H36 and H38 yielded genomes with very low quality in both comparisons, these samples were excluded from this assessment (Table S2). For the LongAmp-amplified sequences, the number of contigs (contigs ≥0 bp) ranged from 102 to 293, and the contig N50 length ranged from 4.6 to 61.0 kb. The mismatches per 100 kb ranged from 325 to 1,285. When reference genomes were included, a genome fraction (total number of aligned bases in the reference genome, divided by the genome size) between 41.9% (sample H25, *S. uberis*) and 84.9% (sample H34, *E. faecium*) was observed .

Concerning the MDA-amplified sequences, the number of contigs (contigs ≥0 bp) ranged from 1 to 56, and contig N50 length ranged from 14 to 2,322 kb. The mismatches per 100 kb ranged from 355 to 1,502. When reference genomes were included, a genome fraction between 35.3% (sample H34, *C. kroppenstedtii*) and 88.1% (sample H22, *S. haemolyticus*) was observed. For samples H25 and H31, a genome fraction below 5% was only observed.

### Short-read metagenomics

#### Illumina pre-assembly quality and host depletion assessment

Here, we assessed the use of MDA to enrich reads of microbial sources following DNA extraction with ML. Samples without pre-MDA treatment were also included for comparison. Samples H29 and H39 replaced samples H36 and H38 in the group of samples that underwent MDA treatment because of their limitation on the amount of DNA. A total of 236.6 million reads were obtained after trimming and filtering. By adopting bmtagger to perform *in silico* separation of bacterial reads from bovine sequences, our results indicate that samples pre-treated with MDA have much a higher percentage of microbial reads (median, 91.1%; mean, 70.6%) than host sequences when compared to not treated samples (median, 1.7%; mean, 3.6%).

#### Illumina post-assembly assessment

Using the assembler metaSPAdes incorporated in the MetaWRAP pipeline, the metagenomic data of all samples treated with MDA achieved a total assembly length of 34.6 Mb, whereas untreated samples reached a total of only 7.4 Mb. When reference genomes were included, it was possible to observe a high genome coverage (observed here as mean genome fraction in percentage) from MDA use compared to untreated samples (*C. kroppenstedtii* with MDA: 98.4%, without MDA: 9.2%; *E. faecium* with MDA: 87.3%, without MDA: 86.8%; *S. chromogenes* with MDA: 92.8%; without MDA: 1.2%; *S. epidermidis* with MDA: 92.1%, without MDA: 0.6%; *S. haemolyticus* with MDA: 88.6%, without MDA 87.8%; *S. hominis* with MDA 89.7%, without MDA 0.2; *S. uberis* with MDA 99.5%, without MDA 52.7%).

**Fig 7:**
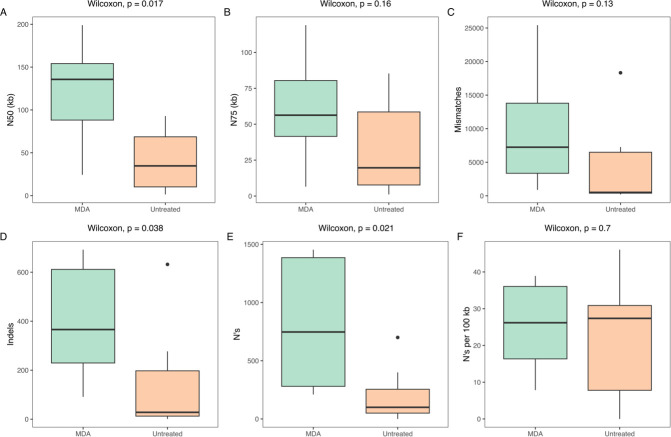
Post-assembly quality control of short-read sequencing data for milk samples collected from individual quarters with high somatic cell count. The median value is shown as a line within the box. Whiskers extend to the most extreme value within 1.5*IQR. Statistical differences between groups were analyzed with the Wilcoxon test (*n* = 6/group).

We evaluated a total of six parameters (Fig. 7) to assess the impact of using or not using MDA on the assembly and quality of contigs. The use of MDA significantly increased the N50 of the contigs compared to the untreated group. It is also possible to observe a significant increase in insertions, deletions, and ambiguous bases. There were no significant differences for the N75, mismatches, and ambiguous bases per 100 kb.

### Metataxonomic analysis

We obtained a total of 5.30 × 106 high-quality filtered sequences (431 bp, percentile 98%) from 38 samples enrolled in this study, and the number of sequences ranged from 68,332 to 334,876 per sample (median frequency: 124,443 sequences; mean frequency: 139,495 sequences).

**Fig 8:**
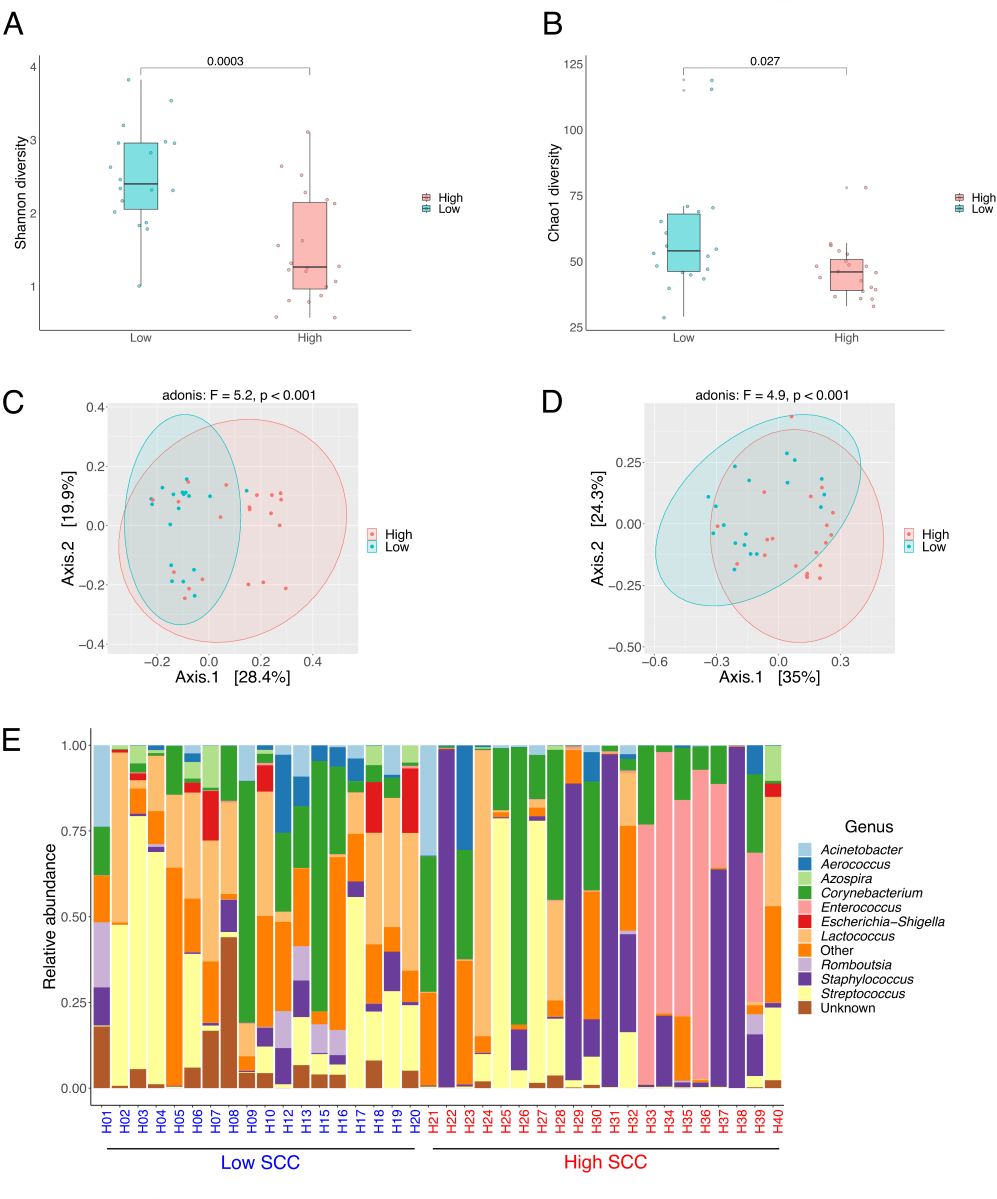
Box and whisker plots comparing Shannon diversity (**A**) and Chao1 diversity (**B**) between samples with low (*n* = 18) and high SCC (*n* = 20). Horizontal bold lines show the median values. The bottom and top of the boxes show the 25th and the 75th percentiles, respectively. The whiskers extend up to the most extreme points within 1.5*IQR. Principal coordinate analysis based on unweighted (**C**) and weighted (**D**) UniFrac distances for the two different groups. Stacked bar plot based on the relative abundance distribution of major genera (**E**) across the different samples.

Alpha-diversities were compared between the groups by the indices Shannon and Chao 1 (Fig. 8A and B). Both parameters were significantly higher in the group with low SCC when compared to high SCC. The milk bacterial community structure of milk samples with low and high SCC was assessed by the unweighted and weighted UniFrac distance metrics. As depicted in Fig. 8C and D, a scatter plot of the principal coordinate analysis (PCoA) using both distance metrics revealed that both communities differed significantly from each other (Adonis, unweighted UniFrac: *P* = 0.001, F-value = 5.2; Adonis, weighted UniFrac: *P* < 0.001, F-value = 4.9). Unweighted and weighted UniFrac components (PCoA 1 and PCoA 2) accounted, respectively, for 48.3% and 59.3% of the total variance.

We detected the following phyla in the milk samples analyzed in this study: *Proteobacteria*, *Firmicutes*, *Bacteroidota*, and *Actinobacteria*. At the genus level (Fig. 8E), *Streptococcus*, *Staphylococcus*, *Romboutsia*, *Lactococcus*, *Escherichia-Shigella*, *Enterococcus*, *Cutibacterium*, *Corynebacterium*, *Aerococcus*, and *Acinetobacter* account for the most abundant taxa.

**Fig 9:**
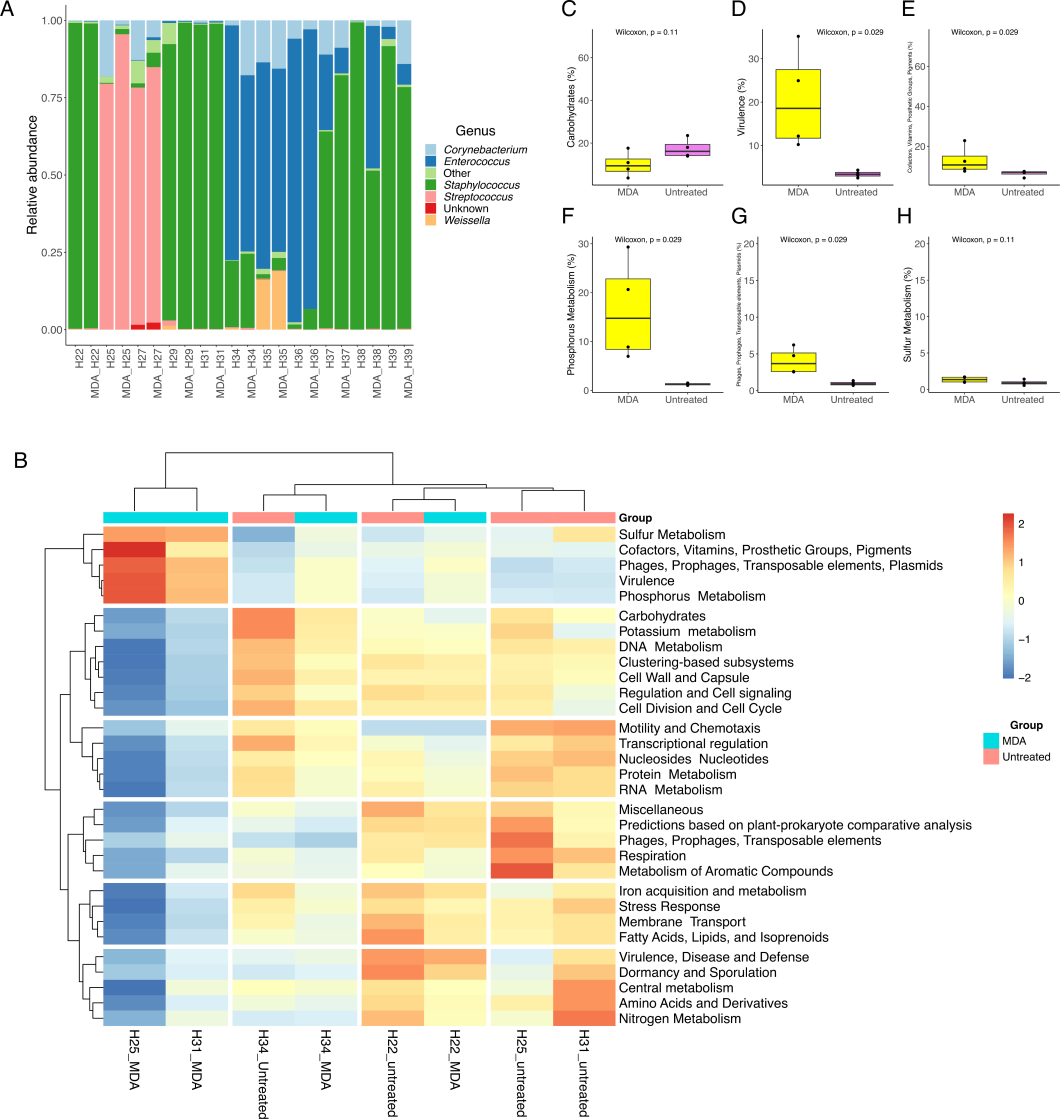
Milk microbial composition based on 16S rRNA gene amplicon sequencing (**A**) of 11 MDA-treated samples and corresponding untreated ones. Functional analysis of shotgun metagenomic carried out with SUPER-FOCUS at subsystem 1 based on the relative abundance of 31 subsystems (**B**). Box plot was constructed based on the relative abundance of six subsystems of interest (**C**). The median value is shown as a line within the box. Whiskers extend to the most extreme value within 1.5*IQR. Statistical differences between groups were analyzed by ANOVA followed by Tukey’s test (*n* = 4/group). Higher values correspond with greater abundance (red).

We also evaluated whether the use of MDA might exhibit a bias toward specific taxa at the level of amplicon sequencing. For this analysis, 11 samples extracted with ML were used and selected based on (i) high somatic cell counting and (ii) positive for a specific mastitis-causing agent. As depicted in Fig. 9A, *Corynebacterium, Enterococcus, Staphylococcus*, and *Weissella* accounted for the most abundant genera. Samples that underwent MDA treatment kept the same microbial composition when compared to the untreated ones, evidencing that no bias toward any bacterial taxa has been introduced. Except for sample H38, where an increase in the abundance of *Enterococcus* following MDA treatment was observed, there were only minor shifts in terms of relative abundance across the different taxa. The use of MDA had no impact on the alpha-diversity indices Shannon and Chao1 (Fig. S8).

### Genome-resolved metagenomic data analysis

We first compared the microbial composition and its functional annotation between MDA and untreated samples to evaluate whether MDA introduced any bias that could have skewed species abundance distribution. Based on MetaPhlAn3, short-reads from shotgun metagenomic sequencing were assigned to seven bacterial species: *S. haemolyticus, S. uberis, S. chromogenes, C. kroppenstedtii, S. hominis*, and *E. faecium*. Overall, both samples shared the same bacterial species, except for the sample H34_MDA, where 3.86 of the reads were assigned to *C. kroppenstedtii*, not represented in sample H34 (untreated sample) (Table S3). Noticeably, the inclusion of three different negative controls allowed the identification of *Cutibacterium acnes* as a contaminant of reagents and/or kits used in the different steps of microbial DNA extraction.

We also analyzed the impact of using MDA on microbial community functionality with SUPER-FOCUS. As depicted in Fig. 9B, there is a remarkable difference between treated and untreated samples, especially in a cluster of subsystems annotated as “sulfur metabolism”, “cofactors, vitamins, prosthetic groups, prosthetic groups, and pigments”, “phages, prophages, transposable elements, and plasmids”, “virulence”, and “phosphorus metabolism”. Among the subsystems outlined above, the pairwise comparison revealed that MDA treatment significantly increased the annotation of genes associated with virulence, phages, prophages, transposable elements, and plasmids (Fig. 9D and G).

Overall, the bacterial community in the hindmilk proved to be less complex in untreated samples when compared to MDA-treated samples given that only three and seven MAGs were recovered, respectively. All MAGs were high quality (more than 90% complete and less than 5% contaminated) and were taxonomically assigned to three different phyla (*Firmicutes*, *Proteobacteria*, and *Actinobacteria*).

**Fig 10:**
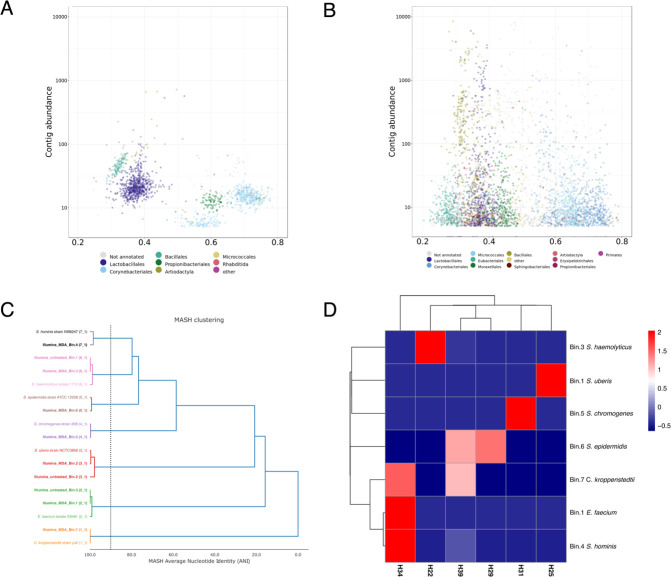
Order taxonomy of the entire assembled community and genome-resolved analysis. (**A**) Guanine-cytosine content (GC) vs abundance plots of contigs from milk samples with high somatic counts without pre-MDA treatment. (**B**) GC vs abundance plots of contigs from milk samples with high somatic counts in MDA-treated samples. (**C**) Average nucleotide identity (ANI)-based phylogenetic tree of 10 MAGs and seven reference strains constructed by MASH clustering (vertical dashed line corresponds to a threshold of 90%). Numbers between parenthesis indicate the secondary cluster. (**D**) Heatmap constructed based on MAG abundance across the different milk samples. Higher values correspond with greater abundance (red).

When contigs were analyzed at the order level, members of *Lactobacillalles*, *Corynebacteriales*, *Bacillales*, *Proprionibacteriales*, and *Micrococcales* were identified in untreated samples (Fig. 10A). In contrast, when samples underwent MDA treatment, members of nine different orders were noticed, namely, *Lactobacillalles*, *Corynebacteriales*, *Bacillales*, *Proprionibacteriales*, *Micrococcales*, *Eubacteriales*, *Moraxellales*, *Sphingobacteriales*, and *Erysipelotrichales* (Fig. 10B).

In total, we were able to reassemble the genome of seven different species in MDA-treated samples, namely, *S. haemolyticus*, *S. uberis*, *S. chromogenes*, *S. epidermidis*, *Corynebacterium kroppenstedtii*, *E. faecium*, and *Staphylococcus hominis*, whereas only *S. haemolyticus*, *S. uberis*, and *E. faecium* were recovered and reassembled from untreated samples. As depicted in Fig. 10C, pairwise comparison in terms of average nucleotide identity (ANI) grouped MAGs and their correspondent reference genomes in the same cluster assuming a 90% MASH ANI threshold, which reinforces their taxonomical assignment with GTDB-Tk.

We further quantified and evaluated the abundance of each MAG for MDA-treated samples across the six different milk samples (H22, H25, H29, H31, H34, and H39) chosen for metagenomic shotgun sequencing. Our results indicate that the genome of each mastitis-associated pathogen initially identified through a culture-dependent technique could be recovered and reassembled following MDA treatment. As shown in Fig. 10D, it is possible to verify the prevalence of single species in four out of six samples (H22, H25, H29, and H31). However, for two samples, the scenario of a co-infection by the microorganisms *C. kroppenstedtii*/*E. faecium*/*S. hominis* (H34) and *C. kroppenstedtii*/*S. epidermidis*/*S. hominis* (H39) was observed and not detected by the official mastitis laboratory report.

## DISCUSSION

Mastitis is the result of a host response to eradicate invading microorganisms, particularly from bacterial sources, and represents a major health concern and cost to the dairy industry (31, 32). While somatic cells are a useful indicator used in the dairy industry to determine raw milk quality (33), the presence of bovine DNA in metagenomic studies of the milk microbiome is undesirable and still represents a major challenge to overcome. The establishment of protocols for microbial DNA extraction of milk samples and bioinformatic pipeline for the reconstruction of MAGs by directly analyzing the genetic material of complex microbial communities will improve pathogen detection, contribute to animal health, and strengthen bacterial diagnosis. This will allow for the surveillance of antimicrobial resistance genes in bovine herds and molecular epidemiology.

In this study, several objectives were set to achieve the best protocol for bovine DNA removal associated with the enrichment of nucleic acid of bacterial (20) origin without affecting the diversity indices. For this assessment, we used high-quality milk (SCC: 125,000 cells/mL; total bacterial count [TBC]: 21,000 cells/mL) that meets the requirements for entry into a milk processing plant.

Among the five different approaches evaluated to deplete host DNA or enrich microbial DNA before sequencing, the best results were obtained by combining two extraction kits (ML-NM) with different DNA capture capabilities (spin column-based nucleic acid purification coupled with magnetic based) or only with MolYsis complete5 (ML). Our results are consistent with what is reported in the literature. In a study conducted by Yap et al. (20), ML also outperformed the other two commercial kits, although the authors used NEBNext Microbiome Enrichment (NM) after cell lysis with a DNeasy PowerSoil Pro kit. The lower performance exhibited when PF was combined with NM could be related to the optimal DNA length required as input (minimum 15 kb fragment size of methylated eukaryotic DNA), as reported by reference 34.

When assessed in milk samples with different levels of somatic cells, ML showed a better performance in removing bovine DNA from a milk sample within a range from 100,000 to 200,000 cells/mL. The justification for this relies on the ratio SCC/IBC. In an ongoing survey in our research group, we analyzed the SCC and IBC of 159 hindmilk samples at the quarter level (unpublished data), and samples with high somatic cells had a median of SCC 33-fold higher than bacteria. There are fivefold more somatic cells than bacteria for samples with low SCC, whereas, for samples with medium SCC, the ratio SCC/IBC is one. This finding aligns with the assumptions stated by Ganda et al. (19), where high-quality raw milk contains around 10-fold more bovine somatic cells than bacteria and, therefore, a 10,000-fold higher abundance of bovine DNA than microbial nucleic acid after adjustment for genome size.

The profiling of the microbial composition (using MetaPhlAn3) was obtained for the different extraction kits tested. MetaPhlAn3 is a very sensitive taxonomic sequence classifier for food microbiomes with low complexity and provides an estimation at the species level and shows a low false-positive rate (35). The highest alpha-diversity indices were observed when ML was used, followed by ML-NM and PF-NM. Furthermore, the comparison of the methods used in this study showed significant differences in the taxonomic composition according to the method used. Among the methods utilized, ML and ML-NM kept the mostly common phylotypes identified in bulk tanks in dairy farms in Norway such as members of the genera *Pseudomonas*, *Corynebacterium*, *Lactococcus*, *Aerococcus*, and *Acinetobacter*, whereas species of the genus *Rhodococcus* were enriched in milk samples extracted with PF. These taxa are also reported in a longitudinal study conducted by Porcellato et al. (36) to evaluate long- and short-term shifts in microbial composition in bulk tank milk using metataxonomic analysis. Likewise, Ganda et al. (19) observed a pronounced increase in the number of microbial reads following PMA treatment; however, a remarkable change in microbial profile was noticed even between replicates of the same biological samples. In this study, PMA treatment negatively impacted DNA quality and, therefore, library preparation. PMA is a DNA-intercalating dye that cannot cross viable cell membranes and, therefore, has been used to discriminate between viable and non-viable bacteria (37). According to Mancabelli et al. (38), the effectiveness of PMAxx in inactivating the free DNA of prokaryotes and eukaryotes tends to vary significantly based on the biological matrices analyzed. The optimization of the method might be needed for a complex matrix such as milk.

In this study, different DNA extraction methods also impacted the functional annotation of the microbial community and the absolute/relative abundance of functional genes. According to Yap et al. (39), genes involved in the metabolism of carbohydrates, proteins, and amino acids and derivatives were identified as the most prevalent in the microbiome of bulk tank raw milk across Ireland, which is in accord with our findings regardless of the extraction method. Overall, the outputs from functional profiling analysis using SUPER-FOCUS are not biased toward the different platforms and depths sequencing (35). However, as reported by McHugh et al. (40), the low number of microbe-associated reads does not allow strain-level classification and, consequently, an in-depth functional classification of the DNA sequences. Taken together, our results suggest that the DNA extraction kit of choice must be considered when analyzing the functional profile of milk samples and comparing it with different approaches.

There is still a gap in the scientific literature concerning the use of long-read sequencing technologies for metagenome studies of milk samples. This is probably because such technologies require as input high-molecular-weight microbial DNA (41), which is seldomly obtained in milk samples. Therefore, to overcome such limitation, we evaluated two different PCR-based methods to amplify microbial DNA sequences from milk samples with high somatic cell count where a mastitis-associated pathogen is present. The protocol described by Alcolea-Medina et al. (42) was developed for viral pathogens and, in our study, adopted for milk samples with low biomass since it utilizes between 1 and 5 ng of input DNA. The protocol uses LongAmp Taq DNA polymerase to amplify fragments up to 30 kb in size. On the other hand, MDA treatment requires between 1 and 10 ng of starting material and is based on the phi29 DNA polymerase. For best results, template DNA should be higher than 2 kb in length with some fragments higher than 10 kb. Both strategies produced reads with a quality score above 7, recommended by ONT (43). However, reads generated with LongAmp had a significantly better Q score on average than those produced with MDA regardless of the sample (LongAmp: 11.1; MDA: 9.7). Reads produced with LongAmp also showed a higher median length when compared to MDA-treated samples. Using both strategies was insufficient to exceed the optimal 10-kb read length commonly observed for genome sequencing of DNA obtained from pure cultures (25).

Regarding metagenomic data assembly, assemblies based solely on long reads produce highly complete and contiguous genomes (44). MDA-treated samples produced less fragmented genomes, estimated by the total number of contigs and N50/N75 values, and similar numbers of mismatches per 100 kb compared to the same samples assembled with sequences derived from DNA amplified with LongAmp. Furthermore, by using MDA, we could cover approximately 35.3% of the *C. kroppenstedtii* genome in sample H34, not identified in the same sample by using LongAmp. As described elsewhere (45), this was most likely due to the positive amplification bias of the LongAmp towards bacterial genomes with lower GC content. Typically, species belonging to the genus *Corynebacterium* have a high GC content (*C. kroppenstedtii*, median GC%: 56.8) (46). The use of MDA was proven efficient for detecting and subtyping *Salmonella enterica* from different food matrices without the need for previous culture enrichment (47). Although polishing tools can be applied on assembled contigs (48) in this study, we chose to keep the original error rate inherent to each protocol before library preparation for comparative purposes.

While we were able to draft three genomes from six individual metagenome assemblies using long read regardless of the PCR-based method (genomes fractions higher to 79.5%), the use of MDA before library preparation followed by short-read sequencing provided better results in terms of recovered draft genomes. In this study, the co-assembly approach was adopted for the assembly of short reads because it has the advantage of recovering low abundant species thanks to the greater sequence depth and coverage by assembling all the samples (49, 50). The best results regarding genome contiguity (N50) and the number of ambiguous bases per 100 kb were obtained with MDA-treated samples. This is because those samples showed on average a much higher amount of high-quality microbial reads when compared to untreated samples. This is probably due to a high DNA fragmentation of the bovine genetic material. Before microbial DNA extraction, a pre-treatment is carried out including the lysis of mammalian cells with a chaotropic buffer followed by nucleic acid degradation with DNase. Larger DNA fragments are necessary for optimal MDA results, and therefore, the presence of microbial DNA with greater fragment sizes can positively favor the PCR toward the amplification of such templates. It should be noted that MDA should be used with caution in metagenomic studies that require quantitative estimation of microbial taxa and functional gene clusters (51, 52).

The increased microbial sequencing depth allowed for further characterization of the microbiome through the generation of seven high-quality draft MAGs in MDA-treated samples, whereas only three MAGs were found in untreated samples. After having analyzed all MAGs following the minimum information about metagenome-assembled genome guidelines (53), comparative analysis clustered all MAGs together with their respective reference aligning at least at 98% ANI, which confirms their species assignments (54). With MDA, we were able to identify two more bacterial species (*C. kroppenstedtii* and *S. hominis* across the samples) that were not initially identified through bacterial isolation and identification with MALDI-TOF. This greatly impacts the inference of antimicrobial resistance genes or mapping metagenomic reads to a database as described by Gweon et al. (55).

In conclusion, this study aimed to evaluate different approaches for host DNA depletion and microbial DNA enrichment from hindmilk. We used commercial kits and coupled them with short- and long-read sequencing technologies and the latest bioinformatics tools to study the microbiome composition and potential functionality by genome-centric metagenomics. Here, we presented several approaches that successfully evaluated the composition and functionality of hindmilk microbiomes by genome-centric metagenomics. We found that it is possible to perform long‐read sequencing with low DNA input and it even achieved a full genome reconstruction of the main pathogen. Based on our results, the application of MDA was superior to LongAmp as (i) it generated less fragmented genomes, (ii) it was unbiased toward microorganisms with high GC content, and (iii) it captured greater diversity of bacterial genera. However, using short-read sequencing and MDA, we were able to recover the high-quality MAGs at higher resolution. Furthermore, this approach found several pathogens in milk samples and was even able to discover an ongoing co-infection not reported by traditional methods for mastitis pathogen identification. The use of this approach can be expanded to improve diagnosis, study the udder microbiome at a greater depth, and detect antibiotic resistance genes. Our results showed that different methods influence t the metagenome composition and functionality, and careful consideration of the results must be considered. While this new approach will improve the detection of mastitis etiology, some limitations are still present such as the long time necessary for DNA extraction, sequencing, and bioinformatics; the cost; and the need for skilled personnel. Furthermore, additional studies are required to investigate the impact of different DNA extraction kits in milk samples from different dairy cow breeds under distinct feeding systems, as well as between foremilk and hindmilk samples.

## MATERIALS AND METHODS

### Study animals and milk sample collection

Fifteen Norwegian Red cows were selected from the “Centre for livestock production” at the Norwegian University of Life Sciences. The farm operates under Norwegian Food Safety Authority regulations regarding food production and animal care. Permission for sample collection and use of information regarding the samples was given by the farm owners. No invasive procedures were used in this study. In this study, dairy cows were housed in freestalls with rubber mats with raw wood chips as bedding materials. Their diet consisted of continuously available silage and pelleted feed based on the milk production of the individual cow. The animals were chosen based on SCC recorded by the automatic milking system (Delaval Online Cell Counter). Seven cows with a low SCC (<100,000/mL: quarters H1 to H20), seven cows with a high SCC (>200,000/mL: quarters H22 to H40), and one with medium SCC (100,000–200,000/mL) were selected for the study.

The aliquot of 1 L of milk was aseptically collected from the bulk tank and divided into 15 Falcon tubes containing 40 mL each. For individual samples, milk samples were collected in 250-mL glass flasks from each quarter, except for three animals, where milk samples from each quarter were pooled together at the same proportion (composite milk) aiming at the assessment of host depletion methods in milk samples with high, medium, and low SCC. Hindmilk was collected at the end of the regular milking routine, as previously described by Porcellato et al. (12). Briefly, after removal of the milking apparatus, the teats were washed with iodine and then ethanol 70%, and 200 mL of milk was collected manually. The “Procedure for Collecting Milk Samples” of the National Mastitis Council (NMC; www.nmconline.org) was followed. After sample collection, the milk was transported on ice until arrival in the laboratory where the samples were immediately frozen at −20°C.

### BacSomatic, culturing, and identification of isolates

Milk samples from all quarters were sent to TINE Mastitis Laboratory, Molde, Norway, for microbiological culture and identification. Bacteriological culturing was performed according to standard procedures (56, 57). In brief, 0.01 mL of milk was spread on cattle blood agar plates with esculin and incubated at 37°C. Plates were read at 24 and 48 h. After the incubation period, species identification was performed with MALDI-TOF MS (Microflex LT system, Bruker Daltonics). Concomitantly, the same samples were tested for SCC and IBC with the instrument BacSomatic (Foss Electric, Hillerød, Denmark).

### Host depletion, microbial enrichment, and metagenomic DNA extraction

For all protocols adopted in this study, a bacterial pellet was obtained from 40 mL of milk sample as previously described by Winther et al. (10), with adaptations. First, 40 mL of milk was thawed on ice and centrifuged at 8,000 ×  *g* for 10 min at 4°C. The whey fraction was discarded, and the bacterial pellet/fat layer was washed in an orbital shaker at 250 rpm for 15 min with a solution containing 2% citrate water and 0.1% Tween 20. After that, the mixture was centrifuged at 8,000 ×  *g* for 10 min at 4°C, the supernatant was removed, and the fat layer was carefully collected with a sterile cotton swab soaked with ethanol 70%. The bacterial pellet was harvested and washed with 2% citrate water (16,200 × *g* for 3 min).

To select the best methods for host DNA depletion and microbial enrichment, a total of five different protocols were assessed as follows.

#### PowerFood

For the mgDNA extraction method with DNeasy PowerFood Microbial Kit (Qiagen, Düsseldorf, Germany), the bacterial pellet was directly transferred to PowerBead tube and processed according to the manufacturer’s recommendations. Aiming for increased efficiency of lysis of difficult species, an additional 5 min of vortex time was added to step 6 of the detailed protocol of the DNeasy PowerFood Microbial kit handbook, adding up to 15 min of vortexing time. The mgDNA was finally eluted in 50 µL elution buffer before storage at −20°C.

#### PMA associated with PowerFood

Bacterial pellet was resuspended in 500 µL Ringer solution (Oxoid, NaCl 2.25 g/L; KCl 0.105 g/L; CaCl_2_·6H_2_O 0.12 g/L; NaHCO_3_ 0.05 g/L). Afterward, 1.25 µL of diluted (1:2) PMAxx (BIOTIUM, California, EUA) was added, and samples were incubated at room temperature for 10 min in the dark. Samples were then treated with PMA-Lite LED Photolysis device for 20 min and then centrifuged at 16,200 × *g* for 3 min. For the mgDNA extraction method with the DNeasy PowerFood Microbial kit (Qiagen, Düsseldorf, Germany), the bacterial pellet was directly transferred to the PowerBead tube and treated as described previously.

#### MolYsis complete5 kit

Bacterial pellet was processed according to the manufacturer’s instructions (Molzym GmBH & Co. KG, Bremen, Germany). Briefly, cells were resuspended in 1 mL of buffer SU, and host cells were lysed by adding 250 µL of a chaotropic buffer (buffer CM), and the released nucleic acids were degraded by an enzyme (MolDNase B). Microbial cells were then sedimented and lysed using reagents and proteinase K. The mgDNA was then isolated and extracted using spin columns, and 50 µL of DNA was eluted and stored at −20°C.

#### PF associated with NEBNext Microbiome DNA Enrichment Kit

The total DNA obtained by adopting PowerFood was treated using the NEBNext Microbiome DNA Enrichment Kit (New England BioLabs, Ipswich, MA) as post-extraction method to remove host DNA, following the manufacturer’s protocol. After the treatment, ethanol precipitation was used to purify the DNA following the NEBNext Microbiome DNA Enrichment Kit’s instructions. DNA was eluted using 50 µL of Tris-EDTA (TE) buffer and frozen at −20°C before further analyses.

#### ML associated with NM

The total DNA obtained by adopting ML was treated using the NEBNext Microbiome DNA Enrichment Kit (New England BioLabs, Ipswich, MA) as a post-extraction method to remove host DNA, following the manufacturer’s protocol. After the treatment, ethanol precipitation was used to purify the DNA following the instructions of the NEBNext Microbiome DNA Enrichment Kit. DNA was eluted using 50 µL of TE buffer and frozen at −20°C before further analyses.

For all individual hindmilk samples with different levels of SCC, a bacterial pellet was obtained from 40 mL of milk sample as described previously, and mgDNA was obtained by using the ML extraction method.

### Multiple displacement amplification by Phi29 DNA polymerase

Following the manufacturer’s recommendation, whole metagenome amplification was performed on 11 samples (H22, H25, H27, H29, H31, H34, H35, H36, H37, H38, and H39) using multiple displacement amplification (MDA) with the REPLI-g Single Cell kit (Qiagen, 150345). These samples were chosen based on their high SCC content and because they covered all the major mastitis-causing pathogens identified across the samples enrolled in this study. In brief, 5 µL of mgDNA was placed into a microcentrifuge tube, 5 µL of buffer D1 was added, and then the mixture was mixed by vortexing. After incubation at room temperature (15–25°C) for 3 min, 10 µL of buffer N1 (stop solution) was added. To the total denatured mgDNA (20 µL), 30 µL of master mix (29 µL REPLI-g mini reaction buffer Solution and 1 µL REPLI-g mini DNA polymerase) was added to the reaction. This solution (50 µL) was gently mixed and incubated at 30°C for 16 h and heat inactivated at 65°C for 3 min (SimpliAmp, Applied Biosystems). The mgDNA concentration was measured by Qubit HS dsDNA assays Fluorometer (Thermo Fischer Scientific, USA).

### ONT library preparation and sequencing

#### Rapid metagenomic sequencing based on MDA and LongAmp

For MDA-treated samples, the SQK-RBK004 rapid barcoding kit was used to prepare the DNA according to the manufacturer’s instructions, including an Ampure XP clean-up step before sequencing. DNA was sequenced on FloMIN 106 R9 version flowcell mk1 with MinKNOW version 18.12.4 according to the manufacturer’s instructions.

Metagenomic assessment based on PCR for milk samples with low bacterial biomass was conducted following the protocol developed by Alcolea-Medina et al. (42), with modifications. This method has been used to perform metagenomic sequencing from extracted DNA and RNA from routine nasopharyngeal swab samples to identify viral pathogens. First, to reach the ideal amount of mgDNA for tagmentation (around 5 ng), samples were up-concentrated by using SpeedVac and resuspended in 3 µL nuclease-free water. Afterward, 3 µL of template DNA was transferred to a 0.2-mL thin-walled PCR tube and gently mixed with 1 µL of fragmentation mix. Samples were then incubated in a thermal cycler at 30°C for 1 min and then at 80°C for 1 min.

For PCR, LongAmp Taq 2× Master Mix (New England Biolabs, NEB M0287) and the SQK-RPB004 rapid barcoding kit were used according to the manufacturer’s recommendations (nuclease-free water: 20 µL; tagmented DNA: 4 µL; rapid barcode primers 01-12A at 10 µM: 1 µL; LongAmp Taq 2× master mix: 25 µL). Amplification was carried out as follows: initial denaturation, 95°C for 3 min (one cycle); denaturation, 95°C for 15 s (30 cycles); annealing, 56°C for 15 s (30 cycles); extension, 65°C for 4 min (30 cycles); final extension, 65°C for 4 min (one cycle).

Following amplification, samples were transferred to individual 1.5-mL Eppendorf DNA LoBind tubes. To each sample, 30 µL of resuspended AMPure XP beads was added and mixed by pipetting. After incubation for 5 min at room temperature on a Hula mixer, samples were pelleted on a magnet, and the supernatant was carefully collected and discarded. Beads were washed twice with 200 µL of freshly prepared ethanol, and after the second washing, samples were allowed to dry for ~30 s and then resuspended in 10 µL of 10 mM Tris-HCl pH 8.0 with 50 mM NaCl. Samples were then incubated for 2 min at room temperature and pelleted on a magnet, and the eluate was collected and transferred to a clean 1.5-mL Eppendorf DNA LoBind tube. All barcoded libraries were quantified by Qubit HS dsDNA assays Fluorometer (Thermo Fischer Scientific, USA) and pooled in a 50–100 fmol ratio in 10 µL of 10 mM Tris-HCl pH 8.0 with 50 mM NaCl. For adapter attachment, 1 µL of RAP was added to the barcoded DNA, mixed gently, and incubated for 5 min at room temperature. The mgDNA was sequenced on FloMIN 106 R9 version flowcell mk1 with MinKNOW version 18.12.4 according to the manufacturer’s instructions.

### Illumina library preparation and sequencing

#### 16 rRNA gene amplicon sequencing

For the metataxonomic analysis of the hindmilk microbiota (sampling occurred at the quarter level), the mgDNA obtained after extraction with the ML extraction method was used. Library preparation for amplicon sequencing using the Illumina MiSeq platform was carried out as described previously (12). In brief, the V3 and V4 regions of the 16S rRNA genes were amplified using the primers Uni340F (CCTACGGGRBGCASCAG) and Bac806R (GGACTACYVGGGTATCTAAT). All PCR reagents and amplification conditions were identical to the one described by Porcellato et al. (12). Negative controls were included to monitor for contamination during DNA extraction and library preparation. Sub-libraries were cleaned and normalized using the SequalPrep Normalization Plate (96) Kit (Thermo Fischer Scientific, USA) and pooled together. The final library concentration was then measured using Qubit 2 with the dsDNA HS kit (Thermo Fischer Scientific, USA) before being sequenced on an Illumina MiSeq platform (Illumina) using the 2 × 300 bp V3 kit (Illumina) at Novogene (Cambridge, UK).

#### Illumina shotgun sequencing

For Illumina sequencing library preparation, the mgDNA concentration of MDA-treated and untreated samples was measured using the Qubit HS dsDNA kit and, when DNA concentration below 0.5 ng/µL was recorded following extraction, an aliquot of 5 μL of the neat sample was used. Otherwise, DNA was diluted to 0.2 ng/µL. DNA was prepared for Illumina sequencing following Illumina Nextera XT Library Preparation Kit guidelines. The DNA concentration was measured by Qubit HS dsDNA assay, and the concentration was then calculated, before diluting and pooling at equimolar ratios. The DNA library was sequenced on the Illumina NovaSeq6000 system (maximum read length of 2 × 150 bp) at Novogene (Cambridge, UK).

### Bioinformatics processing and downstream analyses

#### Long-read quality control, filtering, and metagenome assembly

Adaptor sequences were removed from demultiplexed reads using Porechop v.0.2.4 (https://github.com/rrwick/Porechop) setting up the parameter “--discard_middle.” NanoFilt (v2.8.0) (58) was used to filter the reads with parameters “–l 500 --headcrop 50” as suggested on NanoFilt’s GitHub page. Subsequently, reads were aligned to the bovine genome (RefSeq assembly accession: GCF_002263795.1) using a nextflow pipeline to identify and remove bovine-derived sequences (https://github.com/hoelzer/clean). High-quality and host-depleted reads were assembled with metaFlye v2.8.3 (59) using default parameters. Contig quality was assessed with MetaQUAST v5.0.2 (60) to obtain the overall contig information such as N50, N75, mismatches, number of ambiguous bases, number of ambiguous bases per 100 kb, and indels on the data sets. Taxonomic classification was performed with standard parameters using Kraken2 (61). Seven genomes available in public database (NCBI) were included as references (*Staphylococcus hominis* strain IVB6247, GenBank: CP094724.1; *Streptococcus uberis* strain NCTC 3858, GenBank: LS483397.1; *Staphylococcus haemolyticus* isolate 1710, GenBank: CP113399.1; *Staphylococcus epidermidis* strain ATCC 12228, GenBank: CP043845.1; *Staphylococcus chromogenes* strain 20B, GenBank: CP031471.1; *Enterococcus faecium* isolate E8481, GenBank: LR536670.1; and *Corynebacterium kroppenstedtii* strain yu0, GenBank: CP104321.1). Nanoq (https://github.com/esteinig/nanoq) was used to evaluate the long-read quality (number of reads, number of base pairs, N50 read length, longest read, shorted reads, mean read length, median read length, mean read quality, and median read quality). All bioinformatics analyses were performed with the Orion Cluster HPC at Norwegian University of Life Sciences (NMBU).

#### 16S rRNA gene amplicon sequencing

The demultiplexed raw paired-end reads obtained after sequencing were uploaded and processed into QIIME2 (version 2021.4) via the Casava 1.8 paired-end pipeline (62). DADA2, which allows improved taxonomic resolution based on the exact identification and error correction of sample sequences that differ as little as a single nucleotide, was chosen to assess the quality of the reads in sequential steps such as filtering, trimming, denoising, dereplicating, merging paired reads, as well as chimeric sequences removal (63). Afterward, amplicon sequence variants were forwarded to generate a phylogenetic tree using the align-to-tree-mafft-fasttree pipeline from the q2-phylogeny plugin (64). Taxonomy was assigned to the 16S data using a Naïve Bayes pre-trained silva-138–99-nb-classifier (65).

For downstream metataxonomic analysis, qiime artifacts were imported into R (R Core Team 3.6.2, 2019) with the qiime2R package v.099.20 (https://github.com/jbisanz/qiime2R; accessed on 10 July 2020). Significant differences in alpha-diversity between the groups with high and low SCC were determined using the alpha function in microbiome R package v.2.1.24 adopting Wilcoxon’s test. For beta diversity, weighted and unweighted UniFrac distances were subjected to permutational multivariate analysis of variance (ANOVA) to assess significant differences (pseudo-F test) in bacterial community composition and structure among the groups with a permutation number of 999. PCoA was chosen to explore and visualize the clustering of groups. All graphs were constructed and visualized with RStudio (v. 1.2.5033) as described in “Statistical analysis.”

#### Short-read quality control, filtering, and metagenome assembly

Short reads from the metagenomic data sets were processed through the metaWRAP (66). The quality of the raw sequence data was verified using the FastQC tool (https://www.bioinformatics.babraham.ac.uk/projects/fastqc/). The raw reads were trimmed with Trim-galore v0.4.3 (67), and then, the bovine-derived reads were removed with bmtagger v3.101 (default settings). The high-quality reads were assembled using all six paired-end libraries (co-assembly) per group (MDA treated or untreated) with metaSPAdes v3.11.1 (68). The quality of the generated assemblies was assessed using MetaQUAST v5.0.2 (60). Subsequently, the assemblies were binned with metaBAT v2.12.1 (-m 1500 and --unbinned parameters) (69), Maxbin v2.2.4 (-markerset 40 option) (70), and CONCOCT v0.4.0 (default settings) (71) using the metaWRAP-Binning module. The resulting three bin sets were consolidated with metaWRAP-Bin_refinement, and the completion and contamination of the resulting bins were evaluated with CheckM v1.0.7 (default settings) (72). The taxonomy was assigned to the MAGs using GTDB-Tk v2.2.5 database (73). Only high-quality MAGs (completeness >90%, contamination <5%) were selected for downstream analyses. The abundance of bins across the samples was calculated with the MetaWRAP-Quant_bins module. The taxonomy of each contig was estimated with blastn v2.7.1 (-task megablast -evalue 1e-5 -max_target_seqs 1 -outfmt '6 qseqid sseqid staxids' parameters) with NCBI_nt as the database incorporated in the MetaWRAP-Blobology module. We used dRep (74) for MAGs and reference genomes comparisons. Metagenome functional profile was carried out with SUPER-FOCUS (75), using the aligner DIAMOND (76). All bioinformatics analyses were performed with the Orion Cluster at NMBU.

### Statistical analysis

Statistical analysis and graph construction were performed with RStudio (v. 1.2.5033) using one or the combination of the following R packages: MicrobiomeR (https://github.com/microbiome/microbiome), dplyr (https://github.com/tidyverse/dplyr), ggplot2 (https://ggplot2.tidyverse.org), phyloseq (77), tidyr (https://github.com/tidyverse/tidyr), vegan (https://github.com/vegandevs/vegan), pairwise Adonis (https://github.com/pmartinezarbizu/pairwiseAdonis), and Pavian (78). Groups with parametric distribution were analyzed by one-way ANOVA followed by Tukey’s post hoc. The Wilcoxon test was used to compare two unpaired groups. Differences were considered significant at *P* < 0.05.

## Data Availability

The sequencing data set for this study is available on the Sequence Read Archive (SRA) under BioProject accession number PRJNA950968.

## SUPPLEMENTAL MATERIAL

Supplemental Material msphere.00470-23-s0001.pdf Figures S1 to S8 and Tables S1 to S3.

ASM does not own the copyrights to Supplemental Material that may be linked to, or accessed through, an article. The authors have granted ASM a non-exclusive, world-wide license to publish the Supplemental Material files. Please contact the corresponding author directly for reuse.
