# Supplementary material for: Host DNA depletion methods and genome-centric metagenomics of bovine hindmilk microbiome
Source: mSphere. 2023 Dec 6;9(1):e00470-23. doi: 10.1128/msphere.00470-23 (PMC10826364; doi:10.1128/msphere.00470-23)
Supplement: Supplemental Material — Figures S1 to S8 and Tables S1 to S3. [file msphere.00470-23-s0001.pdf]

Host DNA depletion methods and genome-centric metagenomics of bovine hindmilk microbiome

Vinícius da Silva Duarte[1] & Davide Porcellato[1]

[1] Faculty of Chemistry, Biotechnology and Food Science, Norwegian University of Life Sciences, NMBU, Ås, Norway.

Corresponding author: vinicius.da.silva.duarte@nmbu.no

Supplementary Figure 1. Beta-dispersion (A and B) and beta-diversity (C and D) analyses based on Jaccard (A and C) and Bray-Curtis (B and D) distances for the four different groups. ML: MolYsis complete5 kit; ML_NM: MolYsis complete5 kit performed with NEBNext Microbiome DNA Enrichment kit; PF: DNeasy PowerFood Microbial kit; PF_NM: DNeasy PowerFood Microbial kit performed with NEBNext Microbiome DNA Enrichment kit.

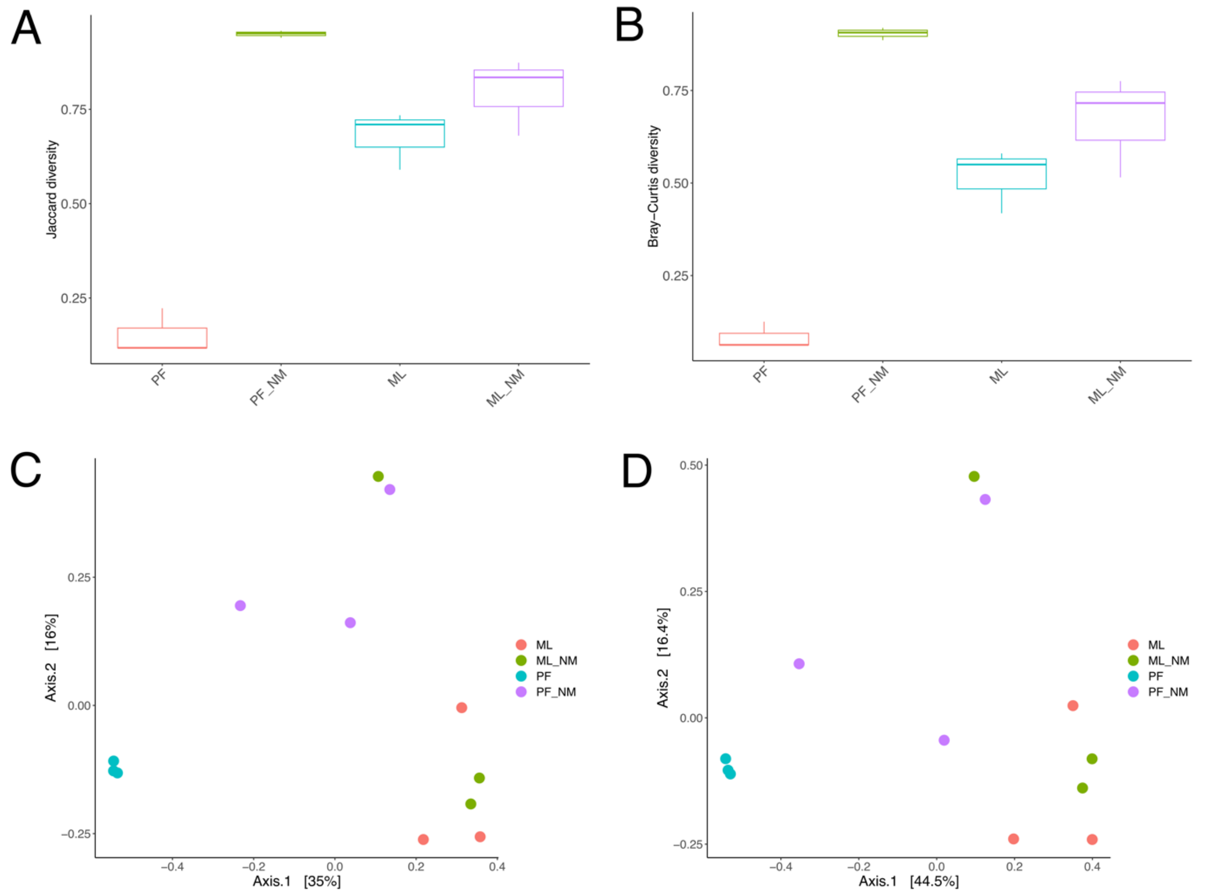

Supplementary Figure 2. Boxplot showing the relative abundance of ten (A-J) of the major bacterial genera identified in bovine raw milk following microbial DNA extraction with four different kits. Statistical differences between groups were analyzed by ANOVA followed by Tukey's test (n = 3/group). Different letters indicate significant differences between groups ($p < 0.05$). ML: MolYsis complete5 kit; ML_NM: MolYsis complete5 kit performed with NEBNext Microbiome DNA Enrichment kit; PF: DNeasy PowerFood Microbial kit; PF_NM: DNeasy PowerFood Microbial kit performed with NEBNext Microbiome DNA Enrichment kit.

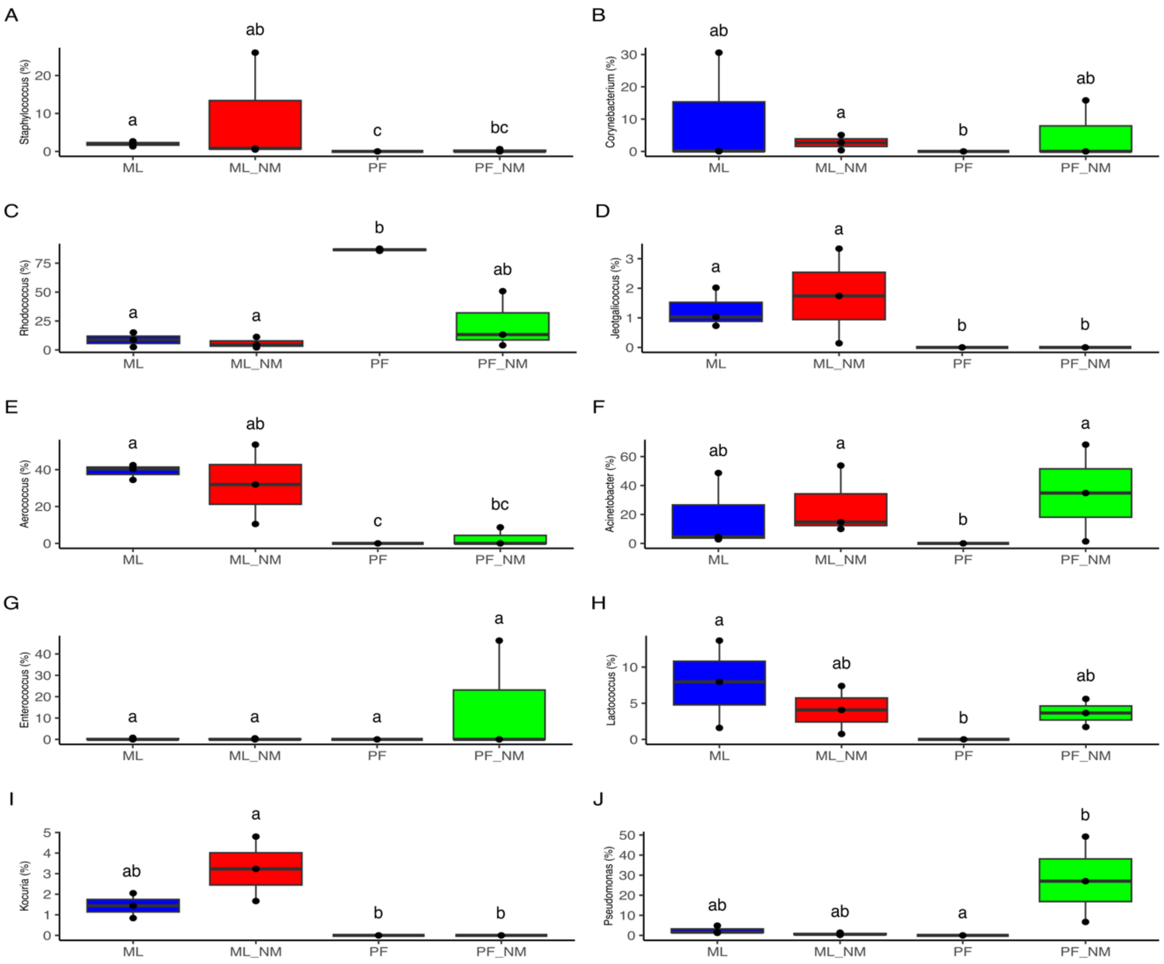

Supplementary Figure 3. Stacked bar plot based on the functional profiles of the bulk tank milk microbiota after DNA extraction and host depletion methods (n = 3/group). A) Absolute abundance of reads assigned to SUPER-FOCUS subsystem level 1 functions**.** B) Relative abundance of reads assigned to SUPER-FOCUS subsystem level 1 functions. ML: MolYsis complete5 kit; ML-NM: MolYsis complete5 kit performed with NEBNext Microbiome DNA Enrichment kit; PF: DNeasy PowerFood Microbial kit; PF-NM: DNeasy PowerFood Microbial kit performed with NEBNext Microbiome DNA Enrichment kit.

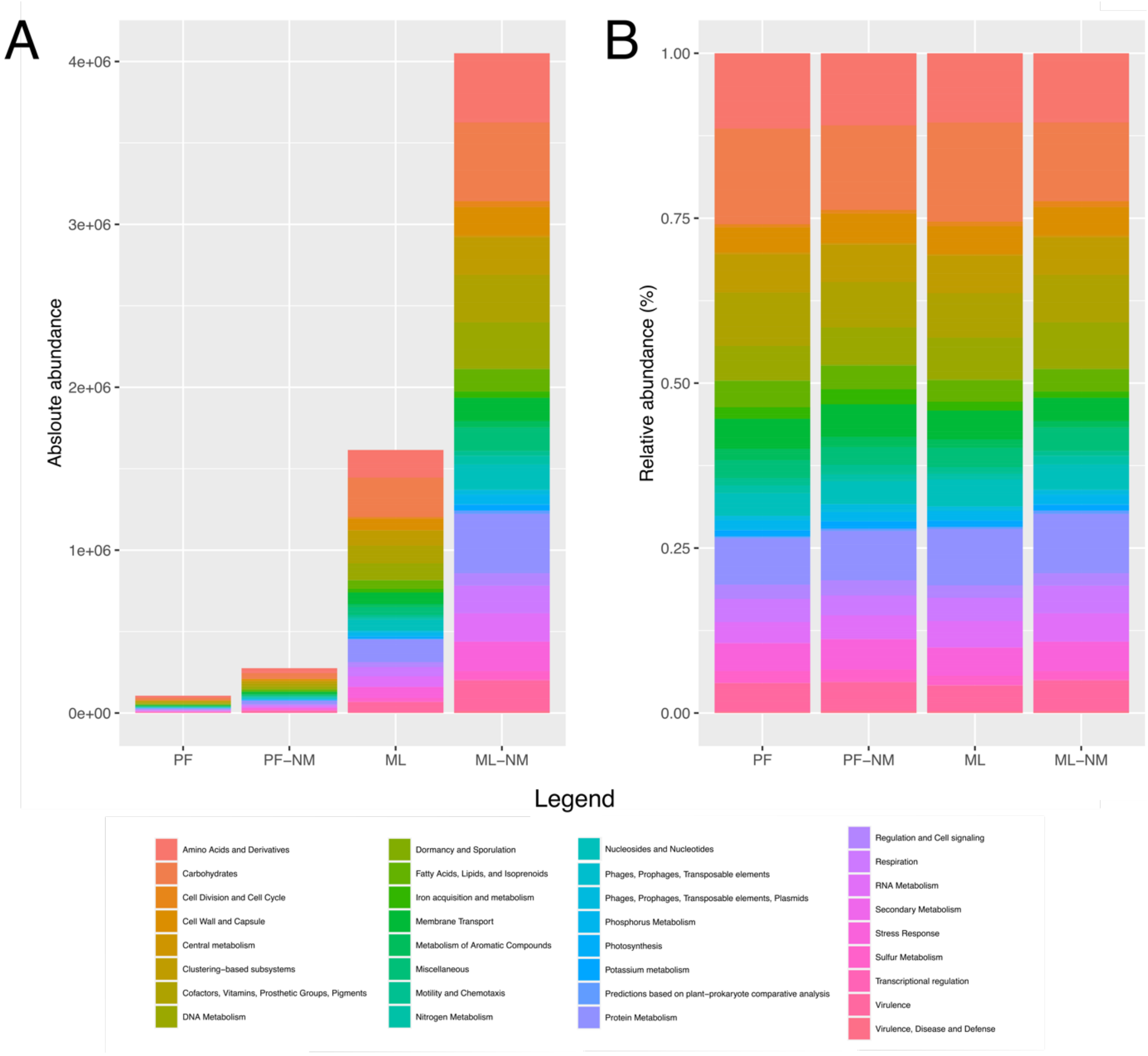

Supplementary Figure 4. Taxonomical assignment of metagenomics reads with Kraken2 after DNA extraction with MolYsis complete5 (n = 6/group). MDA and LongAamp were used to amplify microbial DNA before long-read sequencing.

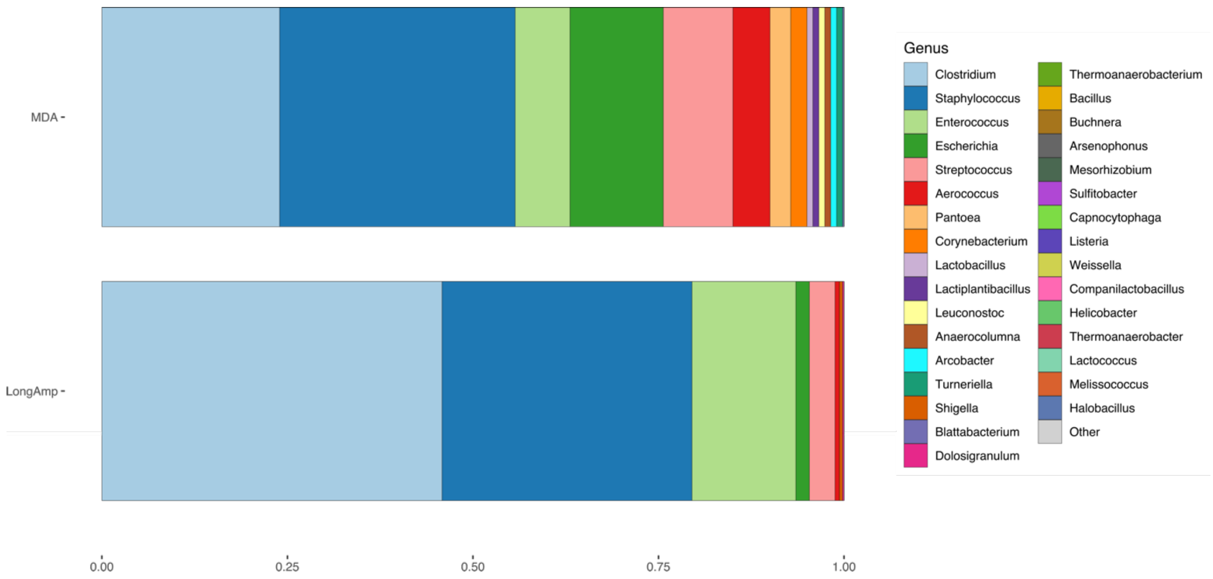

Supplementary Figure 5. Boxplot showing the relative abundance of ten (A-J) major bacterial genera identified in bovine hindmilk samples. Microbial DNA was extracted with MolYsis complete5 followed by amplification with MDA or LongAmp before long-read sequencing. Statistical difference between the groups was analyzed by the Wilcoxon test (n = 6/group).

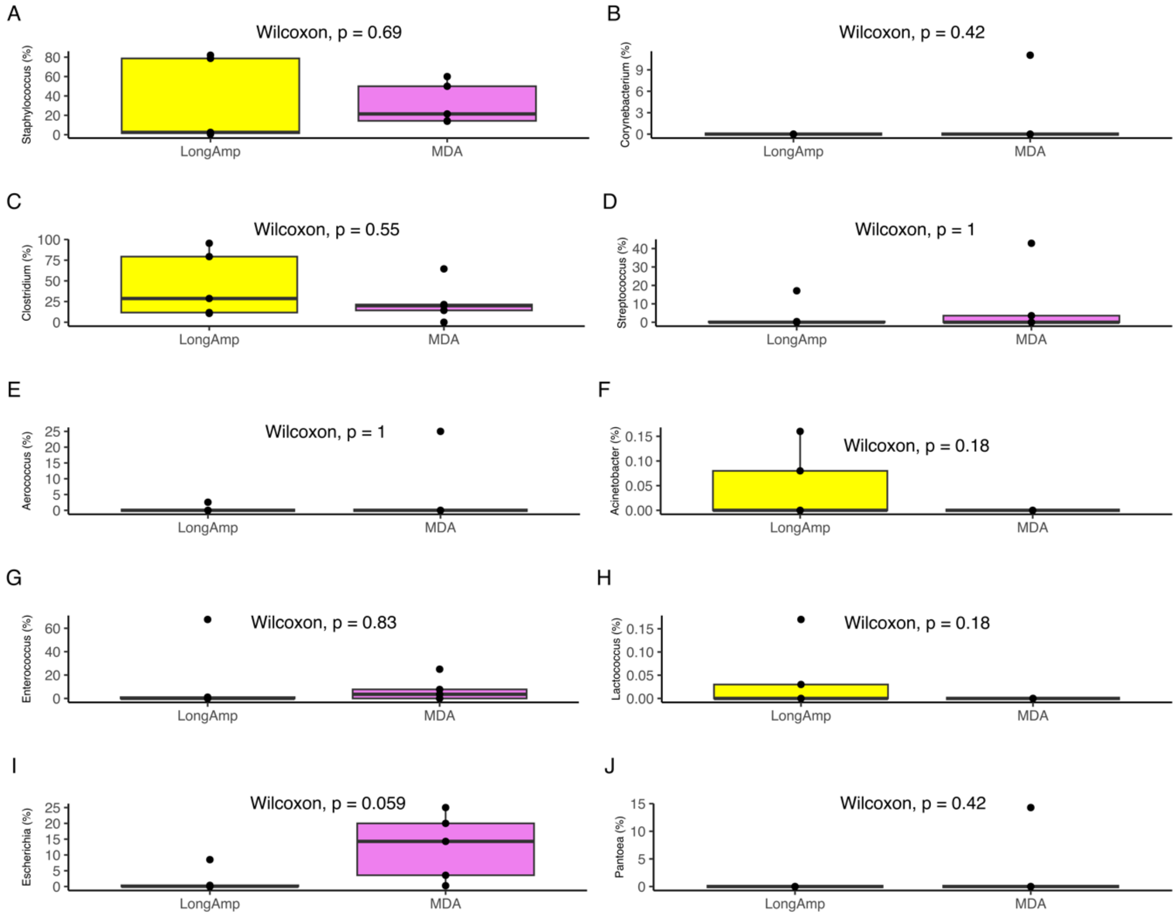

Supplementary Figure 6. Alpha diversity indices were calculated for LongAmp and MDA groups. Plots from A to D represent four different alpha-diversity measures. The median value is shown as a line within the box. Whiskers extend to the most extreme value within 1.5*IQR. Statistical differences between groups were analyzed by the Wilcoxon test (n = 6/group).

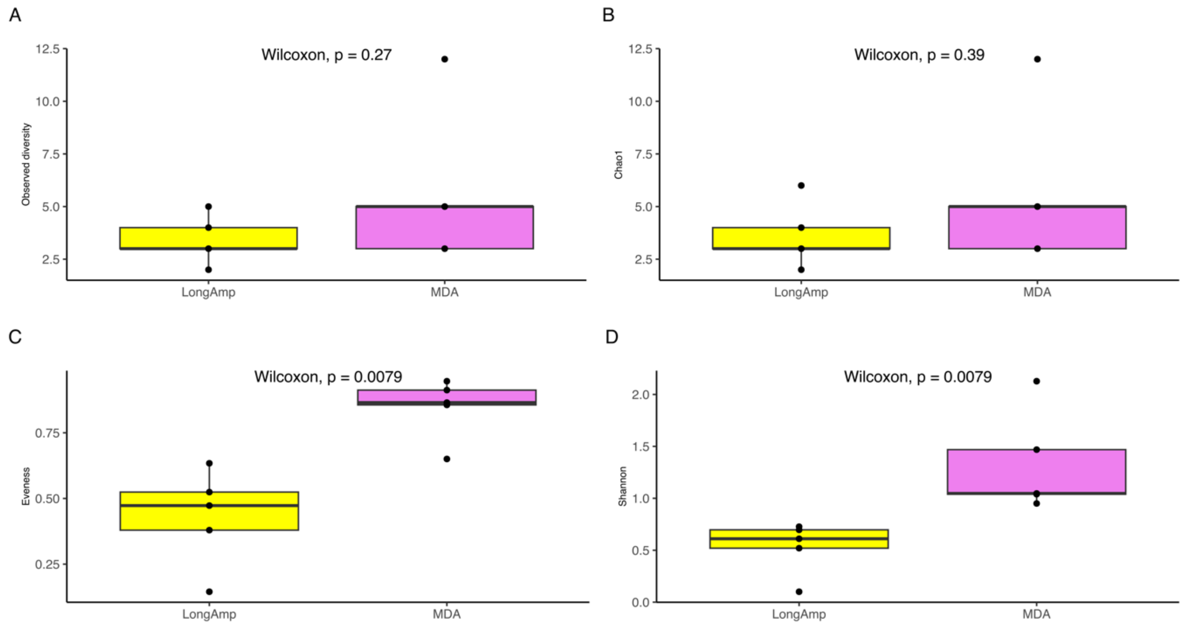

Supplementary Figure 7. Boxplot showing the relative abundance of thirty-tree (A-AI) subsystems (SUPER-FOCUS level 1 functions). Microbial DNA was extracted with MolYsis complete5 followed by amplification with MDA or LongAmp before long-read sequencing. The median value is shown as a line within the box. Whiskers extend to the most extreme value within 1.5*IQR. Statistical differences between groups were analyzed by the Wilcoxon test (n = 6/group).

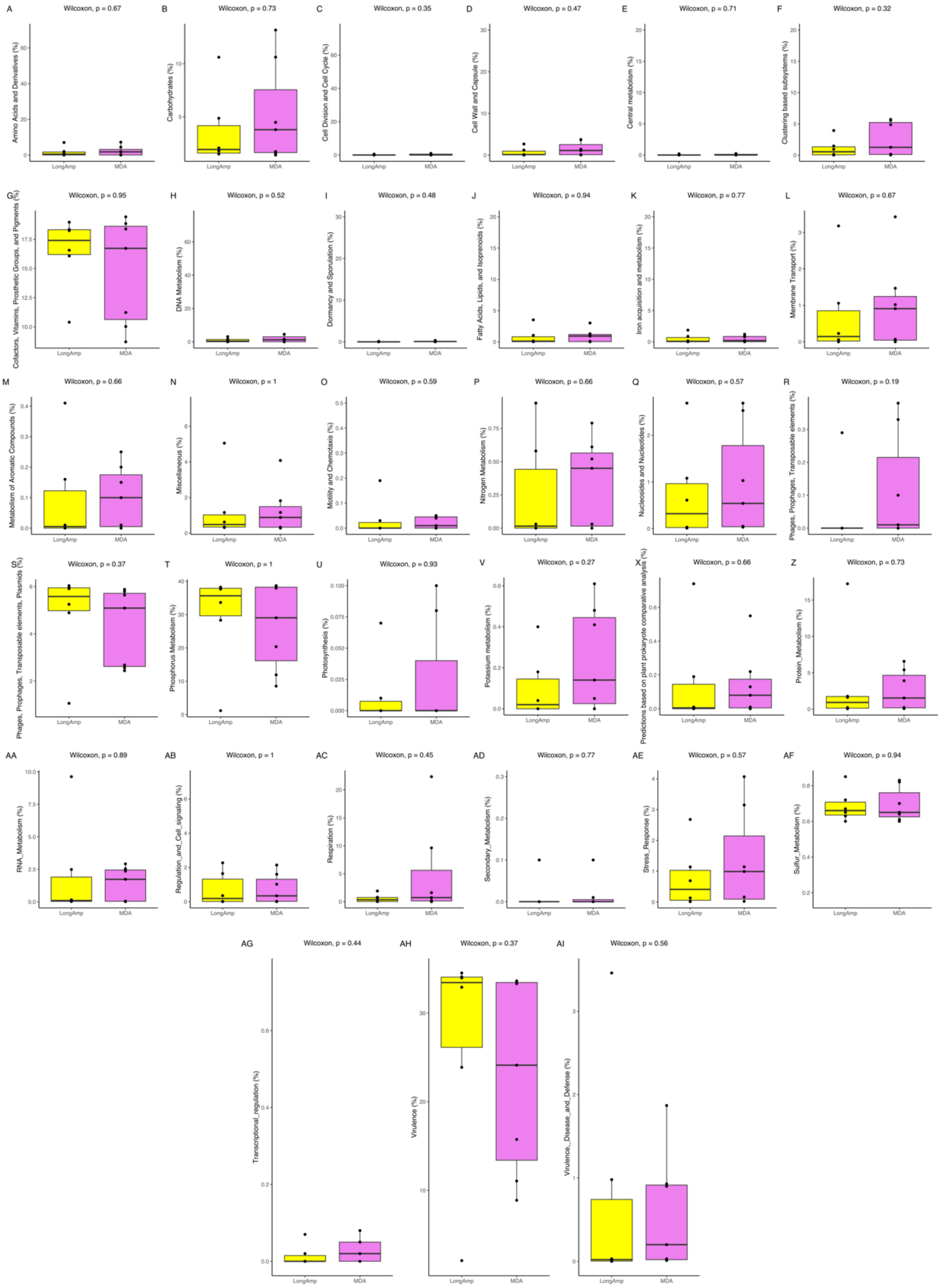

Supplementary Figure 8. Box and whisker plots comparing Shannon diversity (A) and Chao1 diversity (B) between MDA-treated and untreated samples. Horizontal bold lines show the median values. The bottom and top of the boxes show the 25th and the 75th percentiles, respectively. The whiskers extend up to the most extreme points within 1.5 times the interquartile ranges (IQR).

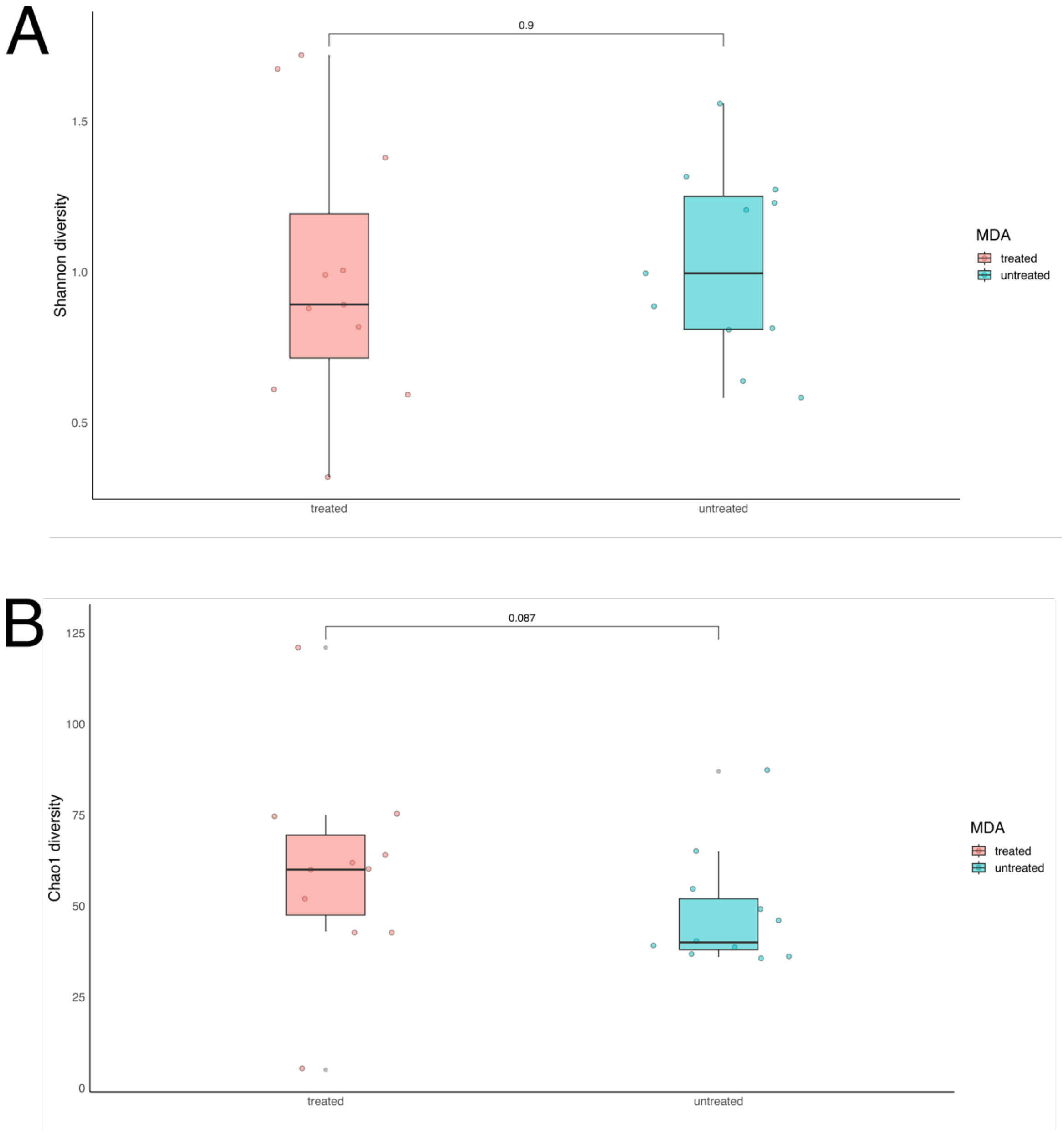

Supplementary Table 1. Statistical analyses of pairwise differences (pairwise-Adonis) in bulk tank milk microbiota. Microbial DNA was extracted with four different kits (n = 3/group). ML: MolYsis complete5 kit; ML_NM: MolYsis complete5 kit performed with NEBNext Microbiome DNA Enrichment kit; PF: DNeasy PowerFood Microbial kit; PF_NM: DNeasy PowerFood Microbial kit performed with NEBNext Microbiome DNA Enrichment kit.

Pairwise Adonis (method: Jaccard)						
	**Pairs**		**F.Model**	**R2**	**p.value**	**p.adjusted**
PF	vs	PF_NM	3.452891	0.4632955	0.1	0.6
PF	vs	ML	9.187646	0.6966858	0.1	0.6
PF	vs	ML_NM	6.591283	0.6223309	0.1	0.6
PF_NM	vs	ML	1.662213	0.2935625	0.1	0.6
PF_NM	vs	ML_NM	1.257252	0.2391462	0.2	1.0
ML	vs	ML_NM	1.103416	0.2162112	0.4	1.0

Pairwise Adonis (method: Bray-Curtis)						
	**Pairs**		**F.Model**	**R2**	**p.value**	**p.adjusted**
PF	vs	PF_NM	3.336187	0.4547576	0.1	0.6
PF	vs	ML	16.298275	0.8029389	0.1	0.6
PF	vs	ML_NM	9.728852	0.7086428	0.1	0.6
PF_NM	vs	ML	2.013405	0.3348194	0.1	0.6
PF_NM	vs	ML_NM	1.515940	0.2748289	0.2	1.0
ML	vs	ML_NM	1.241938	0.2369235	0.2	1.0

Supplementary Table 2. MetaQUAST report of the *de novo* assemblies generated with metaFlye of the six different datasets (H22, H25, H31, H34, H36, and H38) following LongAmp or MDA amplification using ONT reads. Samples H36 and 38 were excluded from this assessment because of the low quality following both procedures.

Flye_LongAmp								
**Sample**	**contigs (>= 0 bp)**	**N50 (kb)**	**N75 (kb)**	**L50**	**L75**	**Genome fraction (%)**	**Total aligned length**	**Mismatches per 100 kbp**
H22	214	9,2	9,2	61	99	79.7 (*S. haemolyticus*)	2108957	820.08
H25	185	5,5	3,9	60	109	41.9 (*Strep. uberis*)	841739	545.01
H31	165	21,6	9,3	34	71	81.4 (*S. chromogenes*)	1837334	1285.73
H34	293	4,6	3,4	94	175	43.2 (*E. faecium*)	1168769	430.93

Flye_MDA								
**Sample**	**contigs (>= 0 bp)**	**N50**	**N75**	**L50**	**L75**	**Genome fraction (%)**	**Total aligned length**	**Mismatches per 100 kbp**
H22	2	2322	2322236	1	1	88.1 (*S. haemolyticus*)	2327897	820.95
H25	3	26	26397	1	1	0.9 (*Strep. uberis*)	25115	3711.31
H31	20	14	8241	6	11	4.9 (*S. chromogenes*)	120563	1254
	56	163	76334	6	3	87.8 (*S. hominis*)	2059203	1502.67
H34	27	272	122461	4	8	87.5 (*E. faecium*)	2379903	355.7
	45	54	26712	9	18	35.3 (*C. kroppenstedtii*)	976311	1133.05

Supplementary Table 3. Taxonomical assignment of metagenomics reads of MDA-treated and untreated samples with MetaPhlAn3.

Species	H22_MDA	H22	H25_MDA	H25	H31_MDA	H31	H34_MDA	H34
Cutibacterium acnes	0,00	0,00	0,00	35,36	0,00	0,00	0,00	0,00
Staphylococcus haemolyticus	99,74	99,78	0,00	0,00	0,00	0,00	0,00	0,00
Streptococcus uberis	0,00	0,00	100,00	63,71	0,00	0,00	0,00	0,00
Staphylococcus chromogenes	0,00	0,00	0,00	0,00	100,00	100,00	0,00	0,00
Corynebacterium kroppenstedtii	0,00	0,00	0,00	0,00	0,00	0,00	3,86	0,00
Staphylococcus hominis	0,00	0,00	0,00	0,00	0,00	0,00	27,29	11,52
Enterococcus faecium	0,00	0,00	0,00	0,00	0,00	0,00	68,85	88,48
